# CRISPR/Cas9 loss-of-function screen in a neuronal model of AP-4 deficiency identifies ATG9A trafficking modulators

**DOI:** 10.1172/jci.insight.202204

**Published:** 2026-05-19

**Authors:** Marvin Ziegler, Cedric Günter, Julian E. Alecu, Xutong Xue, Hyo M. Kim, Afshin Saffari, Alexandra K. Davies, Mustafa Sahin, Darius Ebrahimi-Fakhari

**Affiliations:** 1 Department of Neurology and F.M. Kirby Neurobiology Center, Boston Children’s Hospital, Harvard Medical School, Boston, Massachusetts, USA.; 2 Department of Functional Neuroanatomy, Institute of Anatomy and Cell Biology, Heidelberg University, Heidelberg, Germany.; 3 Laboratory for Axon Growth and Regeneration, German Center for Neurodegenerative Diseases, Bonn, Germany.; 4 Heidelberg University, Medical Faculty Heidelberg, University Hospital Heidelberg, Center for Pediatrics and Adolescent Medicine, Department of Pediatrics I, Division of Child Neurology and Metabolic Medicine, Heidelberg, Germany.; 5 School of Biological Sciences, Faculty of Biology, Medicine and Health, Manchester Academic Health Science Centre, University of Manchester, Manchester, United Kingdom.; 6 Department of Proteomics and Signal Transduction, Max Planck Institute of Biochemistry, Martinsried, Germany.; 7 Rosamund Stone Zander and Hansjoerg Wyss Translational Neuroscience Center, Boston Children’s Hospital, Harvard Medical School, Boston, Massachusetts, USA.; 8 Movement Disorders Program, Boston Children’s Hospital, Harvard Medical School, Boston, Massachusetts, USA.

**Keywords:** Cell biology, Genetics, Neuroscience, Adaptor proteins, Genetic diseases, Neurological disorders

## Abstract

Biallelic loss-of-function variants in adaptor protein complex 4 (AP-4) disrupt trafficking of transmembrane proteins at the *trans*-Golgi network, including autophagy-related protein 9A (ATG9A), leading to childhood-onset hereditary spastic paraplegia (AP-4-HSP). AP-4-HSP is characterized by features of both a neurodevelopmental and a degenerative neurological disease. To investigate the molecular mechanisms underlying AP-4-HSP and identify potential therapeutic targets, we conducted an arrayed CRISPR/Cas9 loss-of-function screen of 8,478 genes, targeting the “druggable genome,” in a human neuronal model of AP-4 deficiency. Through this phenotypic screen and subsequent experiments, key modulators of ATG9A trafficking were identified, and complementary pathway analyses provided insights into the regulatory landscape of ATG9A transport. Knockdown of *ANPEP* and *NPM1* enhanced ATG9A availability outside the *trans*-Golgi network, suggesting that they regulate ATG9A localization. These findings deepen our understanding of ATG9A trafficking in the context of AP-4 deficiency and offer a framework for the development of targeted interventions for AP-4-HSP.

## Abstract 2

A CRISPR drug screen in nerve cells modeling a rare movement disorder identifies two genes whose silencing corrects a key protein trafficking defect.

## Introduction

Approximately one-third of all genes in the eukaryotic genome are predicted to encode transmembrane proteins that must traffic through various cellular compartments to function effectively (1, 2). Defective transport of these proteins has been increasingly linked to a range of neurological diseases, including frontotemporal dementia, Charcot-Marie-Tooth disease, and several types of hereditary spastic paraplegia (HSP) (3, 4). Among these, adaptor protein complex 4–associated (AP-4–associated) hereditary spastic paraplegia (AP-4-HSP) has emerged as a prototypical disorder of impaired protein trafficking. Clinically, AP-4-HSP manifests in early childhood with developmental delay/intellectual disability, progressive spasticity leading to loss of ambulation, epilepsy, secondary microcephaly, and developmental brain malformations (5–8). The condition arises from biallelic loss-of-function variants in one of four genes (*AP4B1*, *AP4E1*, *AP4M1*, and *AP4S1*) encoding the subunits of the heterotetrameric AP-4 complex, which is critical for sorting cargo proteins from the *trans*-Golgi network (TGN) to early and late endosomes and pre-autophagosomal structures (5, 6).

Recent studies have identified autophagy-related protein 9A (ATG9A), the sole transmembrane protein in the autophagic machinery, as an important cargo of AP-4 (9–11). In AP-4–deficient cells, ATG9A accumulates in the TGN and is depleted from peripheral compartments. In neurons, this mislocalization leads to deficits in autophagy, impaired axon maintenance, and length-dependent axonal degeneration (11–16). However, residual autophagic activity and the presence of ATG9A outside the TGN suggest an alternative, AP-4–independent export mechanism (9, 11). This raises critical questions about how ATG9A trafficking is regulated in the context of AP-4 deficiency.

Genome-wide functional genomics screens have emerged as powerful tools to uncover regulators of cellular signaling. However, traditional approaches such as loss-of-function screening using small interfering RNA (siRNA) libraries are often limited by poor reproducibility due to inconsistent gene knockdown and off-target effects (17, 18). Advances in CRISPR/Cas9–based gene interference, particularly its application in high-throughput screening assays, have addressed many of these limitations, ushering in a new era in functional genomics (19). While several large-scale CRISPR screens have been successfully conducted in recent years — primarily as pooled screens in which cells are bulk-transfected with a single-guide RNA (sgRNA) library, selected for specific phenotypes, and analyzed using next-generation sequencing — these approaches pose significant challenges for assessing complex cellular phenotypes (20, 21).

In contrast, arrayed CRISPR screens offer a microplate-based format in which gene modifications are performed individually in each well. This format eliminates the need for complex data deconvolution, facilitates the analysis of intricate readouts such as high-content microscopy, and leverages established platforms for siRNA and small-molecule screening (19). Consequently, arrayed screens provide a robust and scalable approach for functional genomics with broad applications, including drug target discovery (22). However, only a limited number of arrayed CRISPR screens have been conducted to date (23–25).

Here, we present a large-scale CRISPR/Cas9 loss-of-function screen designed to identify modulators of ATG9A trafficking in a neuronal model of AP-4 deficiency, specifically targeting the “druggable genome.” By combining high-content imaging–based multiparametric analysis with a tailored pathway analysis for arrayed screening, we identify novel genes and pathways that modulate ATG9A trafficking. These findings reveal promising targets for drug development and open new avenues for therapeutic interventions in AP-4-HSP.

## Results

### Establishing a phenotypic high-content screen for modulators of ATG9A trafficking.

**Figure 1:**
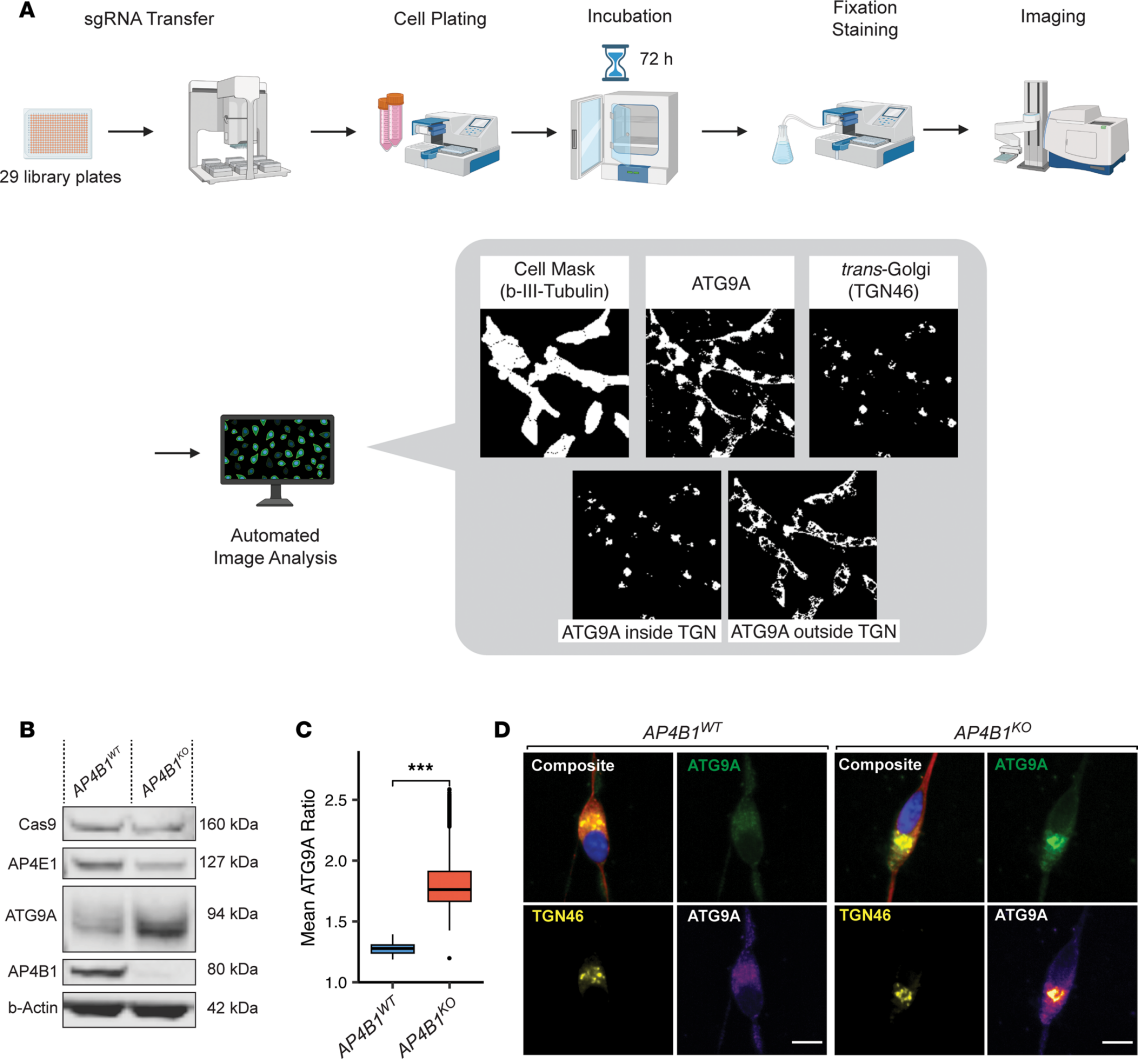
Establishment of a high-content CRISPR/Cas9 loss-of-function screening platform. (**A**) Experimental workflow for CRISPR/Cas9 screen. Control and library sgRNAs were transferred into 384-well plates preloaded with Lipofectamine RNAiMAX. Differentiated Cas9-expressing SH-SY5Y cells were plated on top and incubated with sgRNAs for 72 hours. Cells were subsequently fixed and stained with Hoechst 33258 (nuclear marker), β-tubulin III (cytoplasmic marker), TGN46 (*trans*-Golgi network marker), and ATG9A (ATG9A compartment marker). Plates were imaged using an automated high-content imaging platform (Molecular Devices ImageXpress Micro Confocal Laser System and Peak Analysis & Automation GX robot arm), and images were analyzed using an automated pipeline in MetaXpress to segment cells and calculate the proportion of ATG9A signal overlapping with the TGN. This figure was partially created in BioRender (Kim H, 2026, https://BioRender.com/jrlyt8e). Cellular compartment masks were derived from MetaXpress. (**B**) Western blot analysis of whole-cell lysates from differentiated *AP4B1^KO^* and *AP4B1^WT^* SH-SY5Y cells. *AP4B1^KO^* cells showed loss of AP4B1 protein and reduced levels of AP4E1, indicating successful *AP4B1* knockout and decreased AP-4 complex formation. ATG9A levels were increased in *AP4B1^KO^* cells. Both cell lines demonstrated stable expression of Cas9. (**C**) ATG9A ratio of untransfected *AP4B1^KO^* and *AP4B1^WT^* cells, based on all wells from the primary screen. *AP4B1^KO^* cells showed a significant increase in the ATG9A ratio, consistent with accumulation of ATG9A in the TGN. Statistical analysis: 2-sided Student’s *t* test, ***P < 0.001, Cohen’s *d* ≈ 2.74. (**D**) Representative images of *AP4B1^KO^* and *AP4B1^WT^* SH-SY5Y cells stained with antibodies against ATG9A and TGN46. *AP4B1^KO^* cells exhibited perinuclear accumulation of ATG9A overlapping with the TGN. Pseudocolored images depict grayscale intensity. Scale bars: 10 μm.

We previously developed and validated a high-content imaging–based assay to measure intracellular ATG9A distribution as a surrogate for AP-4 function (26, 27). To identify modulators of ATG9A trafficking in AP-4–deficient cells, we have now leveraged this assay to conduct an arrayed CRISPR/Cas9–based loss-of-function screen (Figure 1A). Given that AP-4-HSP predominantly affects cortical and spinal neurons, we used the SH-SY5Y neuroblastoma cell line, which can be differentiated with retinoic acid into a neuron-like state resembling cholinergic neurons (28). For this study, we used SH-SY5Y cells stably expressing wild-type Cas9, with and without expression of an sgRNA to knock out *AP4B1* (10). Western blot analysis confirmed Cas9 expression and the absence of AP4B1 protein in *AP4B1^KO^* cells, which also displayed reduced levels of AP4E1, consistent with diminished overall AP-4 complex formation. As previously observed in other AP-4–deficient cells (9, 10, 14), ATG9A protein levels were elevated in *AP4B1^KO^* cells (Figure 1B and Supplemental Figure 1; supplemental material available online with this article; https://doi.org/10.1172/jci.insight.202204DS1).

For the CRISPR/Cas9–based loss-of-function screen, SH-SY5Y cells were reversely transfected with the Synthego sgRNA Human Druggable Genome library and incubated for 72 hours. To ensure high consistency and minimize variability, we used automated systems, including sgRNA transfer, pipetting, and liquid dispensing. High-content imaging at ×40 magnification was performed using the ImageXpress Micro high-content imaging system, followed by automated analysis of individual cells with hundreds of cells per well (*AP4B1^WT^*, 930.342 ± 318.332; *AP4B1^KO^*, 845.354 ± 289.564) using a custom pipeline developed with MetaXpress software. Cellular compartments were labeled with 4 markers: Hoechst 33258 (nucleus), β-tubulin III (cytoskeleton), TGN46 (*trans*-Golgi network), and ATG9A (ATG9A compartment). To quantify ATG9A distribution, we calculated the ratio of ATG9A localized inside versus outside the TGN. Consistent with previous findings (9, 10, 26), *AP4B1^KO^* cells showed distinct perinuclear accumulation of ATG9A overlapping with the TGN (Figure 1, C and D), resulting in a significantly elevated ATG9A ratio (*AP4B1^WT^*, 1.276 ± 0.043; *AP4B1^KO^*, 1.817 ± 0.213; *P* < 0.001, Cohen’s *d* ≈ 2.74).

The Synthego sgRNA Human Druggable Genome library targets 8,478 genes, arrayed in a 384-well format, with designated spaces for assay-specific controls. To address potential off-target effects and transfection toxicity, we included negative controls such as *AP4B1^KO^* cells transfected with sgRNAs targeting the non-essential genes *NLRP5* and *KRT77*, as well as untransfected *AP4B1^KO^* cells. Positive controls included *AP4B1^WT^* cells, either untransfected or transfected with sgRNAs targeting *NLRP5*. Additionally, *AP4B1^KO^* cells transfected with sgRNAs targeting the essential genes *COPA* and *KIF11* served as controls to assess transfection efficiency. sgRNAs targeting *ATG9A* in *AP4B1^KO^* cells were used to evaluate the assay’s sensitivity to dynamic changes in ATG9A trafficking (Supplemental Figure 2A).

**Figure 2:**
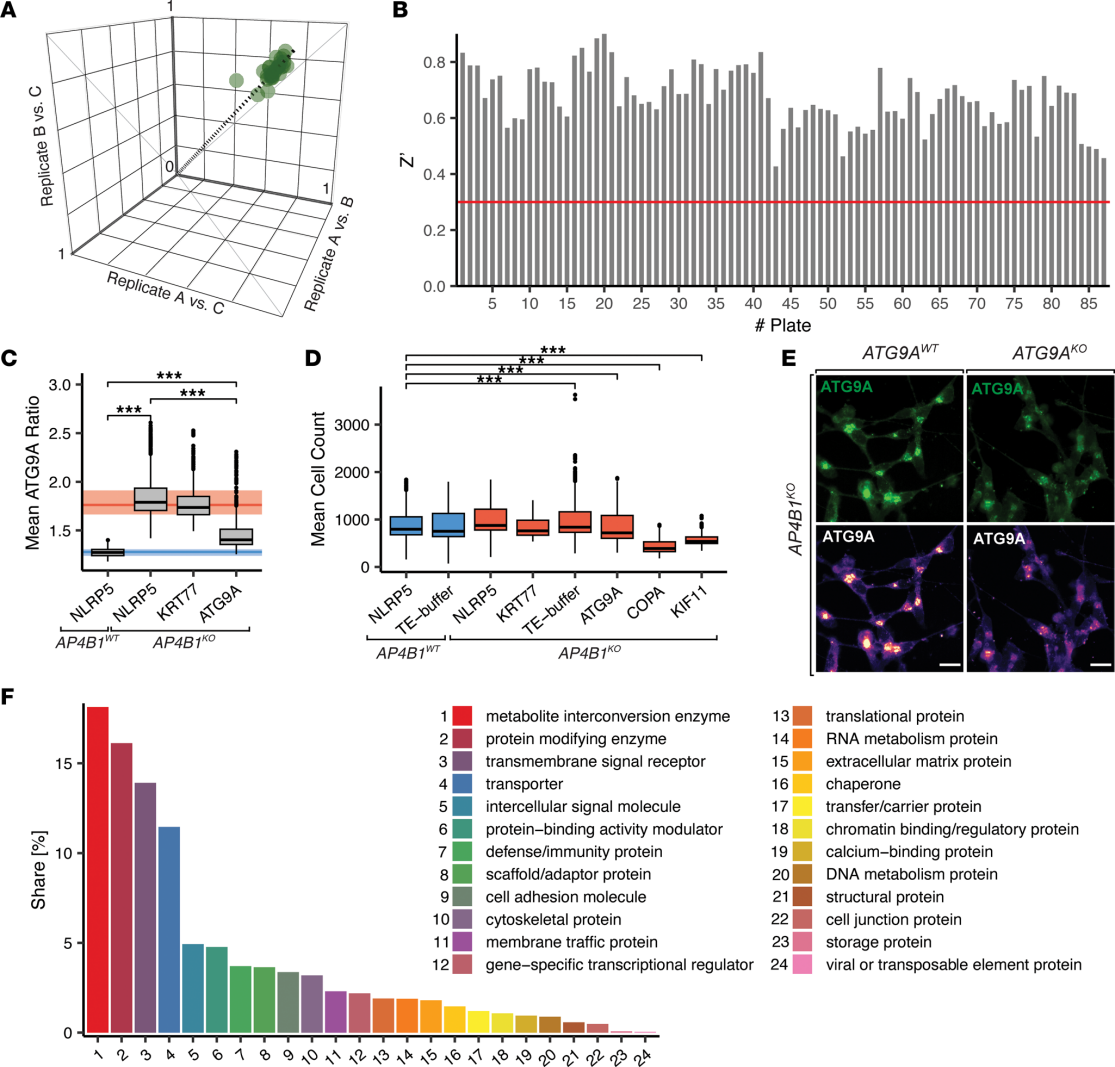
Quality metrics for high-throughput screen and sgRNA library. (**A**) Pearson’s correlation coefficients between replicates across all plates in the primary screen. High correlation between replicates demonstrated low interplate variability. Mean Pearson’s correlation coefficient: 0.907 ± 0.035. (**B**) Robust Z′ for all 87 screened plates confirmed reliable assay performance. Horizontal line indicates predefined quality control threshold (Z′ > 0.3). (**C**) ATG9A ratio for *AP4B1^KO^* and *AP4B1^WT^* cells transfected with sgRNAs targeting non-essential genes (*NLRP5* and *KRT77*), *ATG9A*, and essential genes (*COPA* and *KIF11*). Red line and area represent the ATG9A ratio ± SD for untransfected *AP4B1^KO^* cells (negative control), and blue line and area represent the same for *AP4B1^WT^* cells (positive control). Statistical analysis: 1-way ANOVA, *F*(5) = 1,908, *P* < 0.001, η^2^ = 0.58. Tukey’s honestly significant difference post hoc test revealed significant differences between groups, ***P < 0.001. (**D**) Mean cell counts for *AP4B1^KO^* (red) and *AP4B1^WT^* (blue) cells, including untransfected controls (tris-EDTA [TE] buffer) and cells transfected with sgRNAs targeting non-essential (*NLRP5*, *KRT77*) and essential (*COPA*, *KIF11*) genes, as well as ATG9A. Transfection with sgRNAs targeting essential genes significantly reduced cell counts, confirming efficient transfection and gene knockout. Statistical analysis: 1-way ANOVA, *F*(7) = 163, *P* < 0.001, η^2^ = 0.13. Dunnett’s post hoc test identified significant group differences, ***P < 0.001. (**E**) Representative images of *AP4B1^KO^*/*ATG9A^WT^* (top) and *AP4B1^KO^*/*ATG9A^KO^* (bottom) SH-SY5Y cells stained for ATG9A and TGN46. Pseudocolored images depict grayscale intensity. Scale bars: 20 μm. (**F**) Frequency of Panther GO terms associated with genes in the Synthego Human Druggable Genome library.

Each library plate was screened in 3 technical replicates with high consistency across replicates demonstrated by Pearson’s correlation coefficient (0.907 ± 0.035) (Figure 2A). Quality metrics for high-throughput screening, including Z′ and strictly standardized mean difference (SSMD) (robust versions included), exceeded predefined thresholds (Z′ and robust Z′ > 0.3; SSMD and robust SSMD > 5) (Figure 2B and Supplemental Figure 2, B–D). Knockout of non-essential genes did not alter the ATG9A ratio in either *AP4B1^KO^* or *AP4B1^WT^* cells (Figure 2C). However, minor reductions in cell count suggested low levels of toxicity from off-target effects (Figure 2D). Knockout of *ATG9A* similarly reduced cell counts but significantly decreased the ATG9A ratio in *AP4B1^KO^* cells, nearly matching levels observed in *AP4B1^WT^* cells. This reduction was accompanied by diminished perinuclear ATG9A accumulation (Figure 2E). As expected, knockout of essential genes induced apoptosis. For instance, sgRNAs targeting *COPA* reduced cell counts by approximately 52%, reflecting efficient transfection and knockout. Interestingly, *KIF11* knockout decreased cell counts by only 35%, possibly owing to the reduced importance of *KIF11* in postmitotic SH-SY5Y cells (Figure 2D).

Finally, analysis of the sgRNA library using PANTHER Gene Ontology (GO) terms revealed that most targeted genes encode metabolite interconversion enzymes, protein-modifying enzymes, transmembrane signal receptors, and transporters (Figure 2F).

### Primary and secondary screens identify key modulators of ATG9A trafficking.

**Figure 3:**
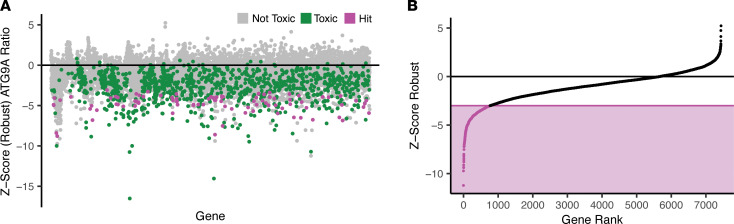
Primary screen identifies modulators of ATG9A trafficking. (**A**) Distribution of robust *z* scores from the primary screen. Genes are categorized as toxic (robust *z* score for cell count < –3 in at least 2 replicates), not toxic, or hits (not toxic and robust *z* score for ATG9A ratio < –3 in all replicates). A total of 92 genes were identified as hits, while 1,041 genes were classified as toxic. (**B**) Ranked distribution of robust *z* scores for all screened genes. Genes with a mean robust *z* score less than –3 across all replicates are highlighted. While only 92 genes met the stringent cutoff criteria of robust *z* scores less than –3 in all replicates and were classified as hits, 764 genes exhibited a mean robust *z* score less than –3 across all replicates, indicating a strong but less reproducible impact on ATG9A trafficking.

As anticipated, the majority of the 8,478 genes screened did not significantly impact ATG9A distribution, as measured by the mean robust *z* score of the ATG9A ratio across all 3 replicates (Figure 3, A and B). The robust *z* score (not to be confused with Z′) is a measure of the effect size calculated from the difference of a given value from the median in relation to the median absolute deviation. Since the screen aims for a reduction of the ATG9A ratio, the greatest negative *z* scores are desired.

We found 1,041 genes (12.28%) that decreased the cell count (robust *z* score < –3 in 2 or more replicates) and excluded these genes from further analysis owing to potential cytostatic or cytotoxic effects. Ultimately, 92 genes met the predefined threshold for ATG9A ratio reduction (robust *z* score < –3 in all replicates), with a mean robust *z* score across all replicates of –4.794 ± 1.123. This represents a hit rate of 1.09%, comparable to other high-throughput screening assays (29).

**Figure 4:**
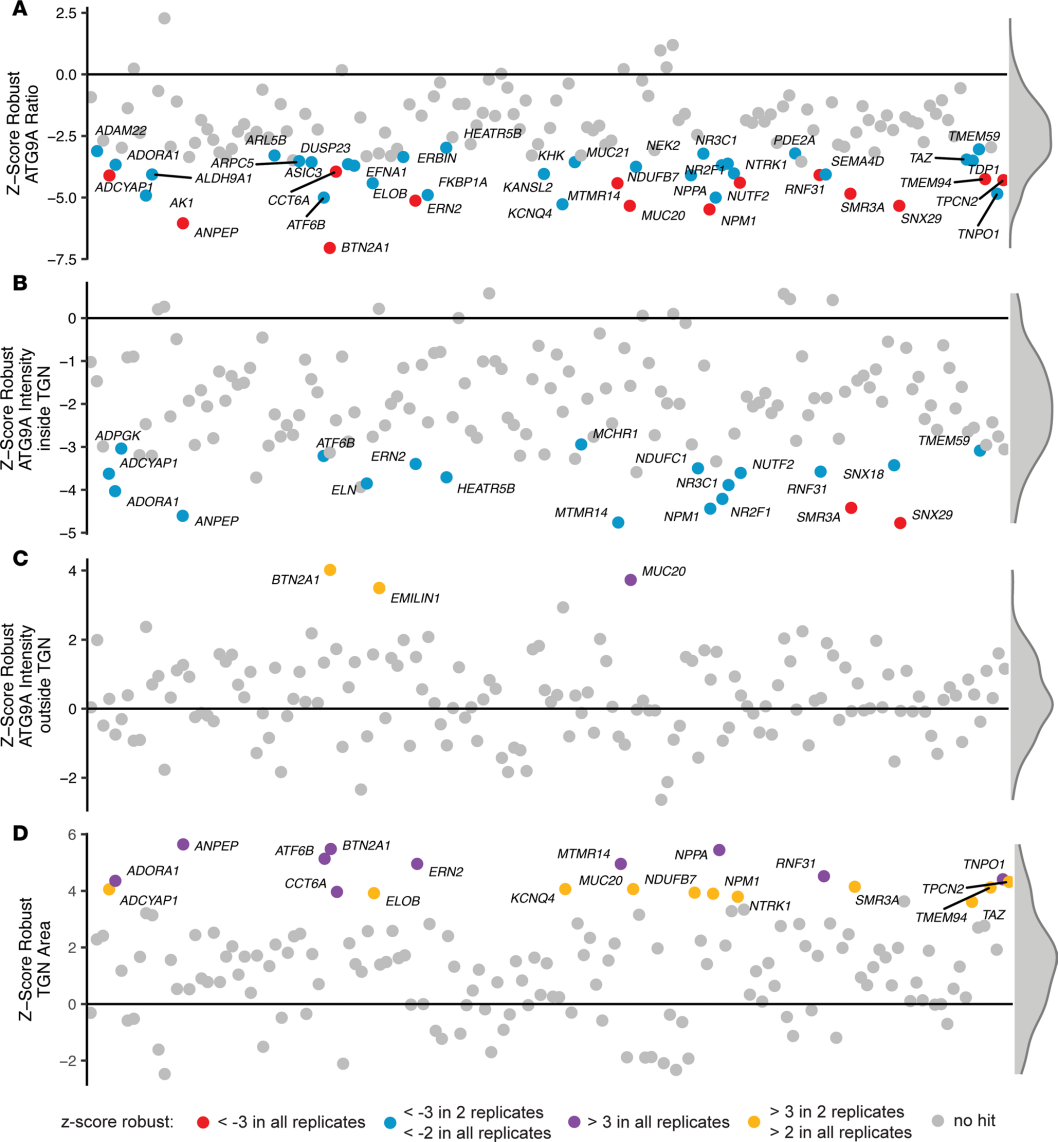
Secondary screen validates modulators of ATG9A trafficking. (**A**) Results of the secondary screen demonstrating robust *z* scores for the ATG9A ratio. A total of 14 genes were confirmed as hits (robust *z* scores < –3 in all replicates); additionally, 30 genes met the less stringent criteria of robust *z* scores less than –3 in 2 replicates and less than –2 in all replicates. (**B**) Robust *z* scores for ATG9A intensity inside the TGN in the secondary screen. Most hits reduced the ATG9A ratio by decreasing ATG9A intensity within the TGN. (**C**) Robust *z* scores for ATG9A intensity outside the TGN. Most genes had minimal impact on ATG9A intensity outside the TGN. (**D**) Robust *z* scores for TGN area. Several genes affected TGN morphology, as evidenced by an increase in TGN area, potentially indicating early toxicity or structural reorganization. Density distribution of robust z scores is displayed on the right in **A**–**D**.

To refine our findings and eliminate false positives, we conducted a secondary screen on the 92 identified hits. An additional 58 genes were selected from the top of the list of genes meeting a slightly less stringent cutoff (ATG9A ratio: robust *z* score < –3 in 2 replicates, robust *z* score < –2 in all replicates, ranked by mean robust *z* score of all replicates) (Supplemental Figure 3A). In rescreening these 150 top-listed genes, we followed the same experimental protocol as in the primary screen but extended the analysis to include a multiparametric TGN morphology characterization, with the goal of identifying confounding effects on Golgi morphology that could impact the ATG9A ratio. Ultimately, 44 genes were confirmed to significantly impact ATG9A translocation: 14 genes consistently met the stringent robust *z* score < –3 threshold across all replicates, while the remaining 30 genes satisfied a robust *z* score < –2 in all replicates and < –3 in at least 2 replicates (Figure 4A).

Most identified genes reduced the ATG9A ratio by decreasing ATG9A signal intensity inside the TGN, with minimal changes to ATG9A levels outside the TGN (Figure 4B). Interestingly, 3 genes — *BTN2A1*, *EMILIN1*, and *MUC20* — primarily increased ATG9A signal intensity outside the TGN (Figure 4C). Additionally, several genes were found to enlarge the TGN area, potentially indicating either a reorganization of the TGN or early signs of cytotoxicity (Figure 4D). The exact biological significance of this TGN enlargement remains unclear and warrants further investigation.

The data from primary and secondary screens are provided as supplemental files (Supplemental Files 1 and 2), offering a comprehensive resource for exploring the genetic modulators of ATG9A trafficking.

### Pathway analysis illustrates modulatory landscape of ATG9A trafficking.

**Figure 5:**
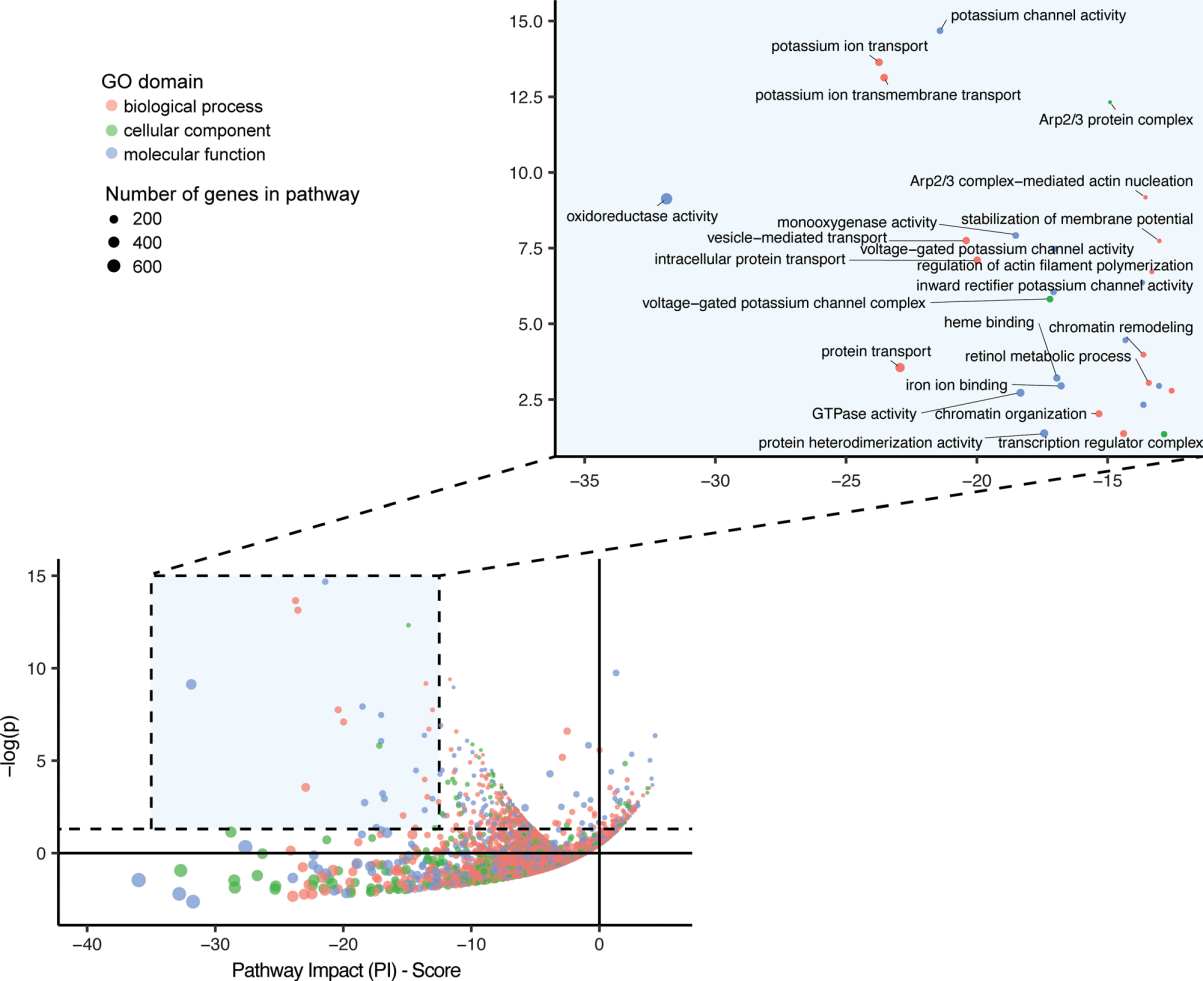
Pathway analysis reveals the modulatory landscape of ATG9A trafficking. Pathway analysis of primary screen results. Each dot represents a GO term. Pathway impact (PI) score reflects the cumulative robust *z* score of all genes associated with the respective GO term adjusted for its size. Dot size corresponds to number of genes linked to the GO term; color indicates Gene Ontology branch. Detailed view of significant pathways with PI scores between –35 and –12.5 is shown on the right, with selected pathways labeled for clarity.

Hit identification in high-throughput screens based solely on ranked readout measures often overlooks the cumulative effects of functionally related genes that do not individually meet hit criteria. To address this limitation, we developed a customized pathway analysis specifically for arrayed high-throughput screens. Unlike conventional pathway analysis tools, our method incorporates the robust *z* score for each gene rather than relying solely on its rank position. Inspired by an approach previously used in siRNA screens (30), we used a model that calculates the cumulative robust *z* score adjusted for the pathway size (pathway impact [PI] score) and determines the likelihood of obtaining a given PI score by chance (*P* value). This model was applied to data from our primary screen. Ultimately, 463 of 15,365 pathways (3.01%) were identified as significantly affected (Figure 5 and Supplemental File 4).

**Figure 6:**
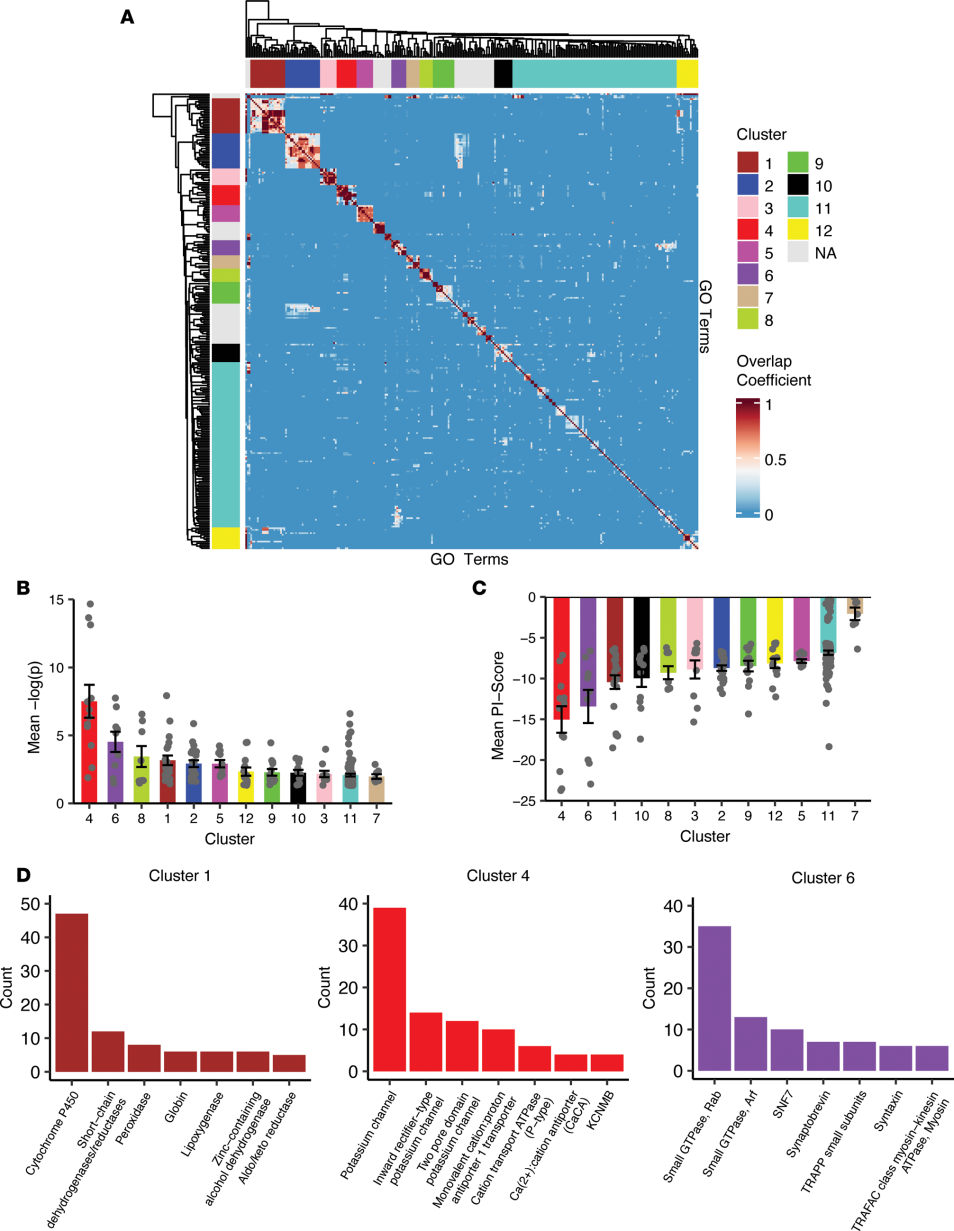
Pathway clustering defines regulatory pathways of ATG9A trafficking. (**A**) Heatmap of genetic overlap between GO terms sorted by average-linkage clustering. Overlap coefficients range from 0 (no genetic overlap) to 1 (complete genetic overlap of the smaller GO term with the larger GO term). Color bars on heatmap edges indicate clusters identified with the Dynamic Tree Cut algorithm. (**B**) Mean *P* values and SD across pathways associated with identified clusters, highlighting their statistical significance. (**C**) Mean PI scores and SD across pathways associated with identified clusters, indicating cumulative impact of genes on ATG9A trafficking within each cluster. (**D**) Protein families and number of proteins from each family associated with top 3 clusters: cluster 1 (oxidative degradation, 192 proteins), cluster 4 (potassium channel activity, 133 proteins), and cluster 6 (vesicular transport, 308 proteins).

To better understand these pathways, we grouped functionally similar pathways into broader clusters by calculating genetic overlaps and constructing a similarity matrix. After average-linkage clustering, we applied the Dynamic Tree Cut algorithm — commonly used in gene expression analysis within the weighted correlation network analysis (WGCNA) package (31, 32) — resulting in 12 distinct clusters (Figure 6A). Each cluster was manually annotated with umbrella terms reflecting its primary function (Table 1). For each cluster, we calculated the mean PI score and mean *z* score (≙ mean *P* value) (Figure 6, B and C). Cluster 4, encompassing GO terms related to potassium channel activity, exhibited the lowest mean *z* score and the highest mean PI score, indicating a substantial impact on ATG9A trafficking. Cluster 1, associated with oxidative degradation, also strongly influenced ATG9A distribution. Notably, cluster 6, comprising GO terms related to vesicular transport, demonstrated significant effects on ATG9A redistribution. Conversely, cluster 11 contained 99 pathways with no meaningful connections and was excluded from further analysis.

To characterize protein families driving these effects, we grouped proteins within the 3 most prominent clusters (lowest PI score) based on their UniProt family annotations. Cluster 1 (oxidative degradation) predominantly featured proteins from the cytochrome P450 family. Cluster 4, consistently the most affected, was enriched with potassium channel family proteins, aligning with the GO terms associated with this cluster. Interestingly, cluster 6, linked to vesicular transport, included primarily members of the small GTPase superfamily, such as Rab and Arf family proteins, but also membrane fusion proteins including synaptobrevin and syntaxin family proteins (Figure 6D). This highlights the important role of small GTPases for ATG9A trafficking in AP-4–deficient cells, something that we have also identified in a previous small-molecule screen (33).

**Figure 7:**
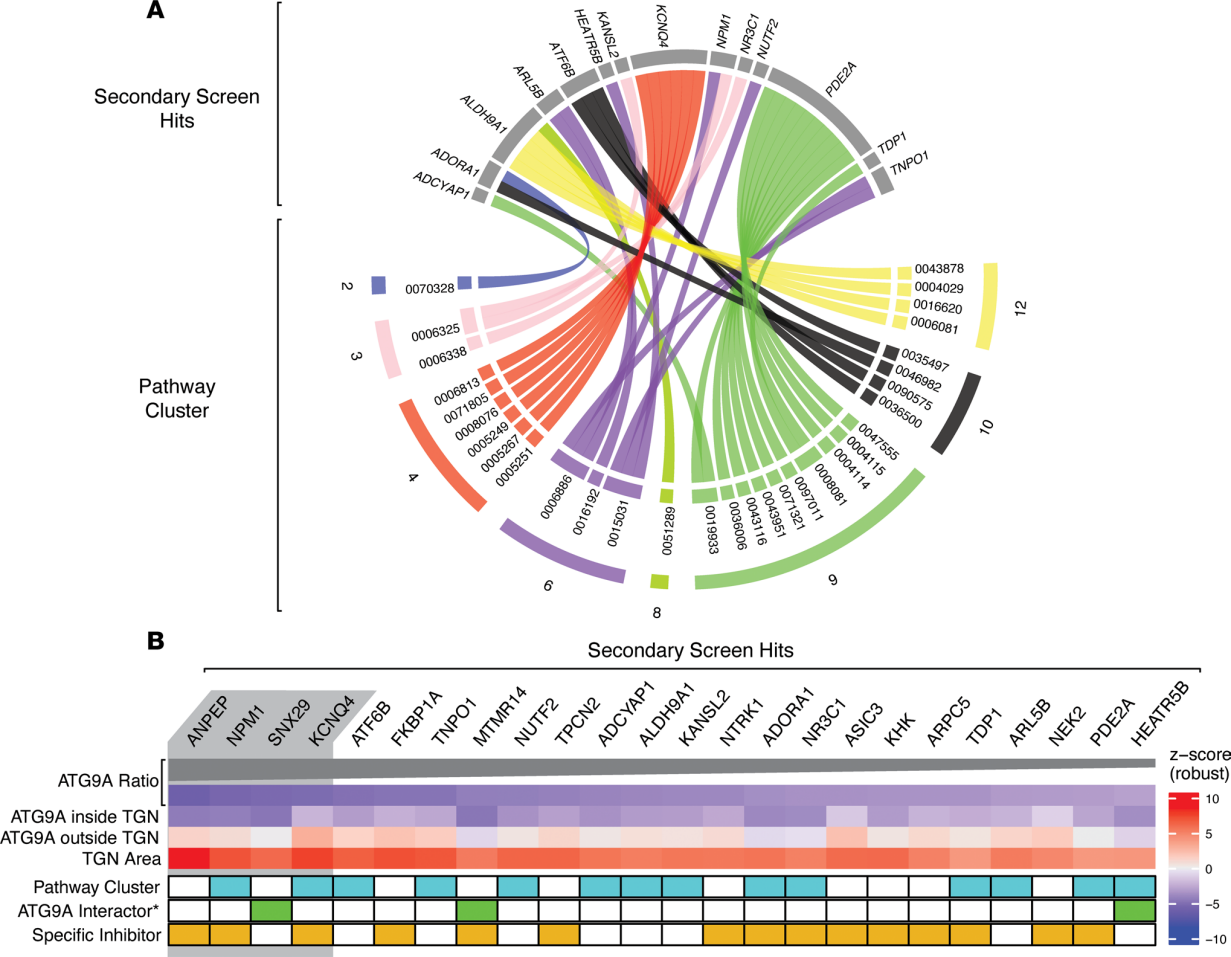
Selection of relevant genes for follow-up. (**A**) Secondary screen hits matched to significantly affected pathways and respective clusters (Supplemental File 5), identifying key modulators of ATG9A trafficking in AP-4–deficient cells. (**B**) Selected secondary screen hits ranked by mean ATG9A ratio reduction across all replicates in the secondary screen and meeting at least one of the following criteria: (a) association with a pathway cluster, (b) identification as an ATG9A interactor by BioID (34), and (c) availability of a specific small-molecule inhibitor. Heatmap summarizes secondary screen results.

Next, we integrated the pathway analysis findings with the results of the secondary screen. Approximately one-third of the confirmed hits from the secondary screen were associated with significant pathways, spanning 8 clusters (Figure 7A). Certain genes were exclusively linked to a single pathway cluster, such as *ARL5B* (“vesicular transport”), *ATF6B* (“cellular stress response”), *HEATR5B* (“vesicular transport”), *KCNQ4* (“potassium channel activity”), *NR3C1* (“histone H3 demethylation”), *NUTF2* (“vesicular transport”), *TDP1* (“signal transduction”), and *TNPO1* (“vesicular transport”). Others, such as *ADORA1* and *NPM1*, were connected to multiple clusters. Given the cellular phenotypes of AP-4 deficiency syndrome, genes involved in vesicular transport (cluster 6) were of particular interest. Notably, *ARL5B*, *HEATR5B*, *NPM1*, *NUTF2*, and *TNPO1* were associated with this cluster. Cluster 2 (“lipid metabolism”) was represented by *ADORA1*.

To corroborate our findings, we compared the hits from the secondary screen with a recent study, which identified ATG9A interactors using quantitative BioID proteomics (34). Remarkably, 3 of our confirmed hits — *HEATR5B*, *MTMR14*, and *SNX29* — were also present in this dataset, highlighting their potential for follow-up studies. Additionally, we evaluated the availability of specific pharmacological inhibitors for the selected genes, facilitating potential clinical translation. We narrowed the list of secondary screen hits to those meeting at least one of the following criteria: (a) association with a relevant pathway cluster, (b) identification as an ATG9A interactor by BioID (34), and (c) availability of a specific small-molecule inhibitor (Figure 7B). The top 4 hits — *ANPEP*, *NPM1*, *SNX29*, and *KCNQ4* — were prioritized for further validation experiments.

### Validation experiments confirm ANPEP and NPM1 as potential therapeutic targets.

To ensure the robustness of our findings from the primary and secondary screens, we performed validation experiments with multiple replicates (Supplemental Figure 4) based on nucleofection, a transfection method that achieves higher knockout efficiencies compared with lipofection used in the earlier screens. Knockout efficiency was confirmed via sequencing and analysis with the ICE (Inference of CRISPR Edits) tool provided by Synthego, which demonstrated knockout scores ranging from 80% to 100%. Moreover, diminished gene expression following knockout of target genes was separately confirmed on the mRNA or protein level (Supplemental Figure 5).

**Figure 8:**
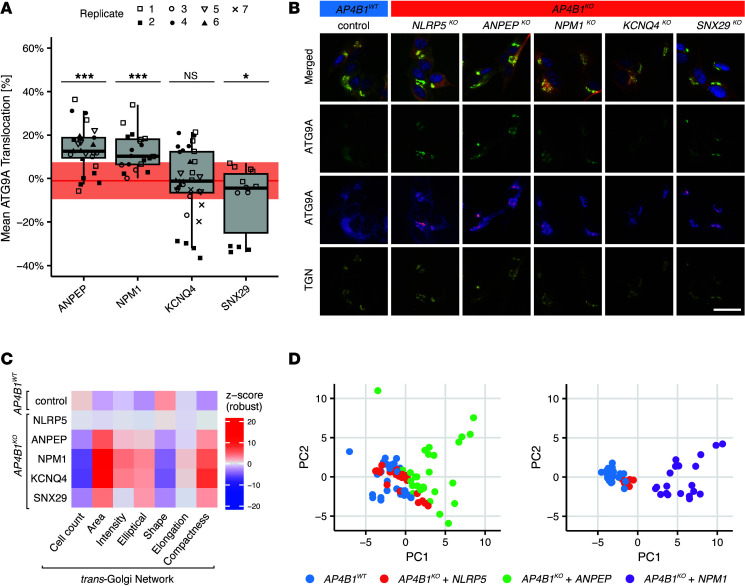
Validation of hits affecting ATG9A translocation. (**A**) Mean ATG9A translocation across up to 7 independent biological replicates after knockout of the top 4 genes (*ANPEP*, *NPM1*, *KCNQ4*, and *SNX29*) compared with knockout of the non-essential gene *NLRP5* (red line and area indicate mean ± SD) in *AP4B1^KO^* cells. Statistical analysis using 1-way ANOVA revealed a significant effect of gene knockout on ATG9A translocation [*F*(5) = 20.48, *P* < 0.001, η^2^ = 0.27]. Dunnett’s post hoc test indicated significant differences in ATG9A translocation for *AP4B1^KO^* + *ANPEP*, *AP4B1^KO^* + *NPM1*, and *AP4B1^KO^* + *SNX29* compared with *AP4B1^KO^* + *NLRP5*. No significant differences were observed between *AP4B1^KO^* + *KCNQ4* and *AP4B1^KO^* + *NLRP5*. Significance level denoted as *P* > 0.05 (ns), *P < 0.05, and ***P < 0.001. (**B**) Representative fluorescence images of *AP4B1^WT^* and *AP4B1^KO^* cells following knockout of *NLRP5* (negative control), *ANPEP*, *KCNQ4*, and *SNX29*. Images highlight differences in ATG9A localization and TGN morphology. Scale bar: 20 μm. (**C**) Heatmap illustrating mean *z* scores for cell count and 6 selected parameters describing TGN morphology. (**D**) Dimensionality reduction of 25 ATG9A-independent cell morphology parameters by principal component analysis (PCA) demonstrated little or no morphological difference between *AP4B1^WT^* and *AP4B1^KO^* cells. Knockout of *NPM1* resulted in a pronounced deviation from controls, while knockout of *ANPEP* caused substantially less morphological changes.

Reevaluation of *ANPEP* (also known as *CD13*) and *NPM1* knockout confirmed their impact on the ATG9A ratio, consistent with the results from the primary and secondary screens. Conversely, knockout of *KCNQ4* and *SNX29* did not demonstrate a similar ATG9A translocation to that seen in both screens (Figure 8, A and B). These findings further support the role of ANPEP and NPM1 in regulating ATG9A trafficking in AP-4–deficient cells. A detailed analysis of various parameters of TGN integrity revealed less pronounced changes upon knockout of *ANPEP* compared with *NPM1* (Figure 8C), suggesting that *ANPEP* plays a more specific role than *NPM1* in ATG9A trafficking without fundamentally disrupting TGN structure.

To assess the broader effects of *ANPEP* and *NPM1* knockouts on cell morphology, we conducted multiparametric profiling using 25 parameters characterizing the cytoskeleton (i.e., cell shape), nucleus, and TGN. Dimensionality reduction by principal component analysis across these cellular morphology metrics revealed substantial differences between the two knockouts. *ANPEP* knockout exhibited considerably less variability in cellular morphology compared with *NPM1* knockout, underscoring the lower toxicity associated with *ANPEP* disruption (Figure 8D). This finding highlights *ANPEP* as a promising target for further therapeutic exploration, with a more favorable toxicity profile than *NPM1*. Raw data for validation experiments are available in Supplemental File 6. 

## Discussion

Despite considerable progress in recent years, the molecular mechanisms underlying AP-4–associated HSP remain incompletely understood. Deciphering these cellular processes is critical for developing new therapeutic strategies for this progressive childhood-onset disease. In this study, we conducted a phenotypic high-content arrayed CRISPR/Cas9–based loss-of-function screen in a neuronal model of AP-4 deficiency, revealing key pathways and identifying modulators of ATG9A trafficking in the context of AP-4 deficiency.

CRISPR/Cas–based functional genomics screens have proven to be a powerful tool for elucidating gene and pathway function. Leveraging the advantages of CRISPR technology over previous genomic screening methods, we interrogated a library targeting approximately one-third of the human genome. To achieve this, we developed an arrayed screening platform using a neuronal cell model of AP-4 deficiency syndrome. By combining this platform with imaging-based multiparametric single-cell analysis, we demonstrated the strengths of arrayed CRISPR screening — offering high accuracy in gene function interrogation alongside a custom experimental and analytical pipeline. A robust assay design, including multiple replicates, stringent controls, and extensive automation, allowed us to maximize the utility of this approach (35).

We acknowledge several limitations of this study. First, SH-SY5Y cells represent a well-established and widely used model for neuronal biology and neurodegenerative disease research. Upon differentiation, their neuronal features are enhanced, including expression of neuronal markers and neurite formation (28), while remaining experimentally tractable for large-scale perturbation approaches such as high-content CRISPR screening. These properties make them particularly suitable for discovery-oriented screening campaigns requiring scalability, reproducibility, and robust phenotypic readouts. However, as a neuroblastoma-derived cell line, SH-SY5Y cells do not fully recapitulate the molecular and functional characteristics of mature postmitotic neurons. More physiologically relevant systems, including neurons derived from induced pluripotent stem cells, may provide complementary platforms to validate and further investigate candidate regulators identified in this screen, although these systems are less amenable to high-throughput approaches.

Second, the alphabetical arrangement of the sgRNA library in the primary screen introduced the potential for plate-based artifacts. To mitigate this risk, we applied stringent quality control criteria and performed a secondary screen with a randomized plate layout, which substantially reduced the likelihood of systematic bias. Third, the transfection efficiency achieved using lipofection may have limited sensitivity, potentially resulting in false negatives for genes with more subtle phenotypic effects. Despite these limitations, our approach identified multiple candidate regulators of ATG9A distribution in AP-4–deficient cells.

Notably, several of the identified genes have established roles in neurological disease. These include *ADAM22*, where biallelic variants are associated with progressive encephalopathy and infantile-onset refractory epilepsy (36); *NR2F1*, where haploinsufficiency is linked to Bosch-Boonstra-Schaaf optic atrophy syndrome (37); *NTRK1*, where biallelic variants are implicated in hereditary sensory and autonomic neuropathy type IV (38); and *TDP1*, associated with spinocerebellar ataxia with axonal neuropathy (SCAN1) (39). Other hits are linked to non-neurological disorders but converge on pathways of potential relevance to neuronal homeostasis. For example, *MTMR14*, which is implicated in centronuclear myopathy (40, 41), functions as a negative regulator of autophagy initiation (42). Pharmacological inhibition of MTMR14 has been shown to enhance autophagic flux and mitigate disease progression in models of neurodegeneration (43).

Consistent with the central role of AP-4 in vesicular trafficking and autophagy, several identified genes are directly connected to autophagic pathways. TMEM59, an endosomal transmembrane protein, promotes selective autophagy by facilitating LC3 recruitment and subsequent lysosomal degradation of endosomal compartments (44). Loss of TMEM59 has been reported to ameliorate disease-relevant phenotypes in a tauopathy mouse model (45). In addition, broader regulators of cellular homeostasis were identified, including ATF6B, a key sensor of endoplasmic reticulum stress within the unfolded protein response (46). Emerging evidence further supports a role for ATF6B in embryonic brain development (47).

Our pathway analysis highlighted several clusters and protein classes as critical modulators of AP-4 deficiency syndrome, offering potential therapeutic targets. As anticipated, proteins involved in vesicular transport emerged as key regulators. Specifically, we identified small GTPases from the Rab and Arf families as essential regulators.

This aligns with previous studies implicating small GTPases in ATG9A and AP-4 cycling. For example, Rab7 has been shown to regulate the recruitment of ATG9A-positive vesicles for mitophagy (48), while Rab1 is essential for autophagosome formation and is present on ATG9A vesicles (49). Beyond this, Rab10 is necessary for ATG9 trafficking in *Caenorhabditis elegans* (50). Our group previously demonstrated that small molecules targeting Rab3C and Rab12 can restore ATG9A distribution in AP-4–deficient cells (33). Arf-like proteins also play crucial roles; for instance, Arl5b regulates retrograde endosome-to-TGN transport (51, 52). In our screen, loss of Arl5b significantly promoted ATG9A redistribution. Potentially, this is mediated by diminished retrograde transport of ATG9A to the TGN.

Another hit, *NR3C1*, encodes a nuclear glucocorticoid receptor and is related to the significant pathway cluster “histone H3 demethylation.” In mouse pancreatic β cells, NR3C1 was found to enhance autophagy via increased expression of the RNA demethylase FTO (fat mass– and obesity-associated protein). FTO catalyzes m^6^A demethylation of *Atg9a*, which promotes mRNA stability and thereby upregulates *Atg9a* expression (53). Consequently, knockout of *NR3C1* in our screen likely lowered *ATG9A* expression and reduced its accumulation in the TGN.

The conservation of AP-4–mediated vesicular transport across eukaryotes is striking; plants exhibit cellular phenotypes similar to those of mammalian cells in the context of AP-4 deficiency (54, 55). In *Arabidopsis* *thaliana* seedlings, AP-4 loss depleted cytochrome P450 proteins (55). In our pathway analysis, cluster 1, dominated by cytochrome P450 enzymes, was among the top 3 clusters, suggesting a conserved role for these enzymes in AP-4–dependent vesicular trafficking. Additionally, lipid metabolism pathways (cluster 2) emerged as relevant. The diverse interconnections between AP-4 and lipid metabolism have been established in both human cells and mouse models, i.e., by the identification of AP-4 cargos such as low-density lipoprotein receptor (LDLR) and diacylglycerol lipase-β (DAGLB) (16, 56). Furthermore, altered trafficking of the AP-4 cargo ApoER2 (apolipoprotein E receptor 2) in AP-4–deficient cells implicated aberrant Reelin signaling (57).

Interestingly, our screen uncovered cation channels, particularly potassium channels, as modulators of ATG9A cycling. Two hits from the secondary screen, *KCNQ4* and *TPCN2*, are prominent representatives. TPC2, a non-selective cation channel localized to endolysosomal membranes encoded by the *TPCN2* gene, is involved in intracellular transport and lysosomal exocytosis, and has been investigated as a therapeutic target for lysosomal storage diseases and neurodegenerative disorders (58). However, KCNQ4, a potassium channel critical for neuronal excitability in cochlear sensory cells (59), together with SNX29, a cellular trafficking protein with a phospholipid-binding domain (60) that has been suggested to be associated with mental disorders (61), showed inconsistent results upon repeated testing. This highlights the challenges of validating subtle phenotypic changes in high-content screens.

Among the confirmed hits, *ANPEP* and *NPM1* emerged as strong candidates for further exploration. Knockout of both genes consistently influenced ATG9A redistribution. However, the impact of *ANPEP* knockout on TGN morphology was less pronounced. This suggested a more specific modulation of ATG9A trafficking, whereas *NPM1* knockout implicated a less targeted effect that resulted in TGN disruption, possibly causing unselective cargo escape. ANPEP, also referred to as CD13, is a membrane-associated protein with aminopeptidase activity. Beyond its enzymatic function, ANPEP exhibits pleiotropic roles and has been described as a “moonlighting protein” (62). It mediates multiple non-enzymatic processes, including regulation of endosomal trafficking and modulation of intracellular signaling pathways such as MAPK, PI3K, and NF-κB (63). In addition, ANPEP has been linked to autophagy through the upregulation of ATG7 expression in human hepatoma cells (64). Its potential involvement in TGN protein export, however, represents a previously unrecognized function. Given its robust effect on ATG9A trafficking and the limited toxicity observed in our system, ANPEP emerges as a candidate for further therapeutic investigation.

In summary, our study provides insights into the mechanisms regulating ATG9A trafficking in AP-4–deficient neuronal cells. We identified candidate genes, such as *ANPEP*, that warrant further investigation as potential drug targets for AP-4-HSP. Additionally, we present a comprehensive dataset for the research community and outline an experimental and analytical pipeline that serves as a blueprint for future arrayed cell-based phenotypic screens.

## Methods

For a table of key resources, please refer to Supplemental Table 1.

### Sex as a biological variable

This study used SH-SY5Y cells, a cell line originally derived from a female patient. Sex itself was not considered a biological variable; we assume that the results of our study are similarly applicable to all sexes.

### Experimental model and study participant details

#### SH-SY5Y cells.

For this study, we used previously published SH-SY5Y cells stably expressing an sgRNA targeting *AP4B1* and Cas9, and SH-SY5Y cells stably expressing Cas9 but no sgRNA (10). Cells were cultured in Dulbecco’s modified Eagle medium/Nutrient Mixture F-12 (Thermo Fisher Scientific) supplemented with 10% fetal bovine serum, 100 U/mL penicillin, and 100 μg/mL streptomycin. Cultures were maintained at 37°C in a humidified incubator with 5% CO_2_. Cells were passaged every 3 days by washing with Dulbecco’s phosphate-buffered saline (DPBS) and dissociation with 0.25% trypsin-EDTA. Changes of medium were performed every other day to ensure optimal growth conditions. For differentiation, SH-SY5Y cells were cultured in neurobasal medium supplemented with 1× B-27 supplement, 2 mM l-glutamine, and 10 μM all-*trans* retinoic acid for 5 days. To improve differentiation and enhance cell attachment, laminin (10 μg/mL) was added to the differentiation medium (65).

#### sgRNA library.

The Synthego sgRNA Human Druggable Genome library was obtained from the ICCB-Longwood Screening Facility at Harvard Medical School. This library targets 8,478 human genes, using 3 distinct chemically synthesized sgRNAs per gene, each designed to target early exons. The library is arrayed across 29 plates in a 384-well format, with designated wells allocated for assay-specific controls to ensure experimental consistency.

### Method details

#### Reverse transfection.

All steps were conducted in an RNase- and DNase-free environment to ensure sample integrity. Positive and negative control sgRNAs (targeting *ATG9A* and *NLRP5*, respectively) were diluted in nuclease-free Tris-EDTA buffer and dispensed into the appropriate wells of a 384-well PCR plate to achieve a final concentration of 50 nM. Lipofectamine RNAiMAX (Thermo Fisher Scientific) transfection reagent was diluted in reduced-serum medium (Opti-MEM, Thermo Fisher Scientific) and distributed into three 384-well assay plates per library plate to create 3 replicates. The final volume of RNAiMAX in the reaction was adjusted to 0.16%. The sgRNA library and control sgRNAs were transferred to the RNAiMAX-containing assay plates using the Agilent Bravo Automated Pipettor and the Agilent 4-R BenchCel Plate Handler. SH-SY5Y cells cultured in differentiation medium for 2 days were counted using an automated cell counter. Cells were then plated at a density of 3,000 cells per well in differentiation medium containing 10 μM all-*trans* retinoic acid, but without laminin. The Multidrop Combi Reagent Dispenser (Thermo Fisher Scientific) was used for dispensing of RNAiMAX solution and cell plating. Plates were spun briefly to ensure even distribution of reagents and incubated at room temperature for 20 minutes to allow uniform cell settlement. The assay plates were incubated for 72 hours at 37°C in a 5% CO_2_ humidity-controlled incubator to minimize evaporation and edge effects. After 24 hours of incubation, 10% differentiation medium supplemented with 40 μM all-*trans* retinoic acid and 10 μg/mL laminin was added to support differentiation and cell adhesion.

#### Nucleofection.

All steps were conducted in an RNase- and DNase-free environment to maintain sample integrity. To enhance gene-editing efficiency, ribonucleoprotein complexes (RNPs) were used for nucleofection. sgRNAs were diluted in tris-EDTA (TE) buffer to a stock concentration of 100 μM, and Cas9 was prepared at a stock concentration of 20 μM. The nucleofector solution was prepared by mixing of the nucleofector reagent and supplement at a 4.5:1 ratio. RNPs were assembled by combining of sgRNA (final concentration 7.2 μM) and Cas9 protein (final concentration 0.8 μM) in the nucleofector solution. Briefly, 180 pmol sgRNA was incubated with 20 pmol Cas9 protein in the nucleofector solution for at least 10 minutes to form RNP complexes. SH-SY5Y cells were harvested, and 4 × 10^5^ cells were resuspended in 5 μL of the prepared nucleofector solution. The cell suspension was mixed with the pre-assembled RNPs at a 1:6 ratio (cell suspension/RNP solution) and transferred into nucleofection strips. The strips were placed in the 4D-Nucleofector System (Lonza), and nucleofection was carried out using the CA-137 program. After nucleofection, pre-warmed medium was added to the cells after a 10-minute incubation at room temperature. The cells were subsequently plated at a density of 1 × 10^4^ cells per well into a 96-well plate using the Integra Voyager 8-channel automated pipettor.

#### Fixation and immunocytochemistry.

After 72 hours of incubation, cells were fixed with 4% paraformaldehyde for 20 minutes at room temperature. Cells were then permeabilized using 0.1% saponin in DPBS for 10 minutes. After permeabilization, the solution was removed, and blocking buffer containing 0.1% bovine serum albumin and 0.01% saponin in DPBS was added for 20 minutes to minimize nonspecific binding. Primary antibodies were diluted in blocking buffer to the following concentrations: rabbit anti-ATG9A (1:500 to 1:1,000), sheep anti-TGN46 (1:800), and mouse anti–β-tubulin III (1:1,000). Cells were incubated with primary antibodies for 1 hour at room temperature and then washed 3 times with blocking buffer. Secondary antibodies were prepared in blocking buffer at concentrations of 1:1,250 to 1:2,000 and included Alexa Fluor 488–conjugated anti-rabbit, Alexa Fluor 594–conjugated anti-sheep, and Alexa Fluor 647–conjugated anti-mouse. Additionally, Hoechst 33258 (1:2,000) was used for nuclear staining. Cells were incubated with secondary antibodies for 30 minutes in the dark, followed by 3 washes with DPBS to remove unbound antibodies. All solutions used for immunostaining were filtered through a 0.22 μM filter system to ensure sterility and consistency. After staining, plates were sealed with opaque seals to prevent light exposure and prepared for imaging.

#### High-throughput imaging.

High-throughput imaging in 384-well plates was performed using the Molecular Devices ImageXpress Micro Confocal Laser System, equipped with a ×40 S Plan Fluor objective (numerical aperture, 0.60 μm; working distance, 3.6–2.8 mm). For each well, 16 fields were acquired in a 4 × 4 grid configuration. For high-throughput imaging in 96-well plates, 24 sites per well were captured in a 5 × 5 grid configuration, leaving out the center position. Sites were spaced 330 μm apart to ensure even coverage of each well. A Peak Analysis & Automation GX robot arm was used for automated plate loading into the microscope. Image analysis was conducted using a customized pipeline within the MetaXpress software (version 6.7.1.157, Molecular Devices). Cells were identified as β-tubulin III–positive areas containing Hoechst-positive nuclei. The *trans*-Golgi network (TGN) was defined by TGN46 signal intensity, while ATG9A protein was identified based on its specific signal intensity. Overlapping TGN46 and ATG9A signals were classified as “ATG9A inside the TGN,” whereas ATG9A signal outside the TGN46-defined region but within the cellular boundary was categorized as “ATG9A outside the TGN.”

#### Western blotting.

Cells were cultured in 6-well plates at a density of 5 × 10^5^ cells per well and harvested using RIPA buffer supplemented with protease inhibitors and phosphatase inhibitors. The total protein concentration was determined using the Pierce BCA Protein Assay Kit with absorbance measured at 562 nm using a Tecan plate reader. Protein samples were adjusted to a total amount of 15 μg, mixed with NuPAGE LDS Sample Buffer, and supplemented with NuPAGE Sample Reducing Agent (Thermo Fisher Scientific). The samples were boiled at 70°C for 10 minutes before loading onto NuPAGE 4%–12% Bis-Tris gels. Electrophoresis was carried out in NuPAGE MOPS SDS Running Buffer for approximately 50 minutes at 200 V. Proteins were transferred onto an Immobilon-FL PVDF Membrane (Millipore) using a wet transfer system with NuPAGE Transfer Buffer at 30 V for 75 minutes. The membrane was then blocked with Intercept (TBS) Blocking Buffer (LICORbio) for 60 minutes on a shaker. Membranes were incubated overnight at 4°C with the following primary antibodies, diluted in Intercept (TBS) Blocking Buffer supplemented with 0.2% Tween 20: mouse anti–CRISPR/Cas9 (1:500) (2-day incubation), rabbit anti-AP4B1 (1:500), mouse anti–β-actin (1:2,000), mouse anti-AP4E1 (1:500), and rabbit anti-ATG9A (1:500). Near-infrared secondary antibodies (IRDye 800CW donkey anti-rabbit IgG; IRDye 680LT donkey anti-mouse IgG, LICORbio) were diluted 1:5,000 in Intercept (TBS) Blocking Buffer supplemented with 0.2% Tween 20 and 0.01% sodium dodecyl sulfate. Membranes were incubated with secondary antibodies for 1 hour at room temperature in the dark. Protein detection and imaging were performed using the LI-COR Odyssey DLx system. Image analysis was carried out using Image Studio software (version 5.2.5, LI-COR).

#### Reverse transcription quantitative PCR.

Total RNA was extracted using the RNeasy Mini Kit (QIAGEN, 74106). cDNA was synthesized using the High-Capacity cDNA Reverse Transcription Kit (Thermo Fisher Scientific, 4368813), following the manufacturer’s protocol. Quantitative PCR was performed using SYBR Green PCR Master Mix (Thermo Fisher Scientific, 4334973). Amplification was carried out on a QuantStudio 6 Flex Real-Time PCR System (Applied Biosystems) under standard cycling conditions. Melt curve analysis was included to verify product specificity.

Ct values were normalized to the mean of the housekeeping gene *RPL32*. Primer sequences are provided in Supplemental Table 1.

#### Available small-molecule inhibitors.

Available small-molecule inhibitors for prioritizing secondary screen hits (Figure 4B) were identified using the International Union of Basic and Clinical Pharmacology/British Pharmacological Society Guide to Pharmacology (https://www.guidetopharmacology.org; accessed February 18, 2025) and the product portfolio of MedChemExpress (https://www.medchemexpress.com; accessed February 18, 2025).

### Statistics

Statistical analysis and plotting were performed using R statistical computing (version 4.2.0) and the RStudio IDE (version 2022.12.0+353). The following R packages were used: dplyr (66), ggplot2 (67), openxlsx (68), ggpubr (69), plotly (70), ggrepel (71), ggExtra (72), janitor (73), lsr (74), tidyr (75), tibble (76), ComplexHeatmap (77, 78), multcomp (79), effectsize (80), bibtex (81), UniprotR (82), dynamicTreeCut (83), ggbreak (84), gggap (85), pbapply (86), cluster (87), purr (88), moduleColor (89), pals (90), Hmisc (91), biomaRt (92, 93), scales (94), RColorBrewer (95), and circlize (96).

For normalization, the robust *z* score was calculated:
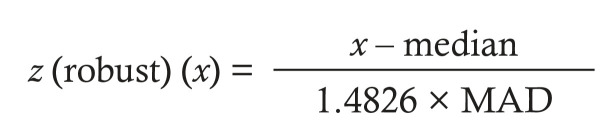
where *x* is value per well, “median” is median of all wells, MAD is median absolute deviation, and 1.4826 is a constant to adjust MAD for asymptomatically normal consistency.

As a quality metric for assay plates to account for plating artifacts and staining irregularities, we calculated the Z′ factor and the SSMD as well as the robust version of both parameters using the median and median absolute deviation:
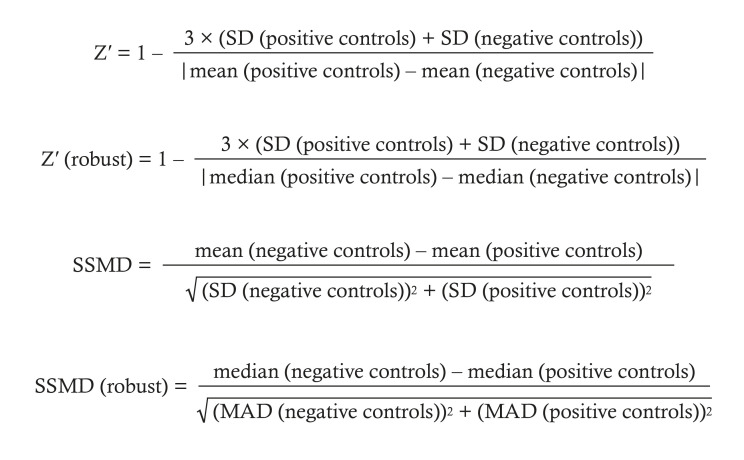


Data are shown as mean ± SD. The Pearson’s correlation coefficient was calculated between all replicates using the *cor()* function in R. Statistical differences between 2 groups were evaluated using 2-tailed Student’s *t* test, with *P* values less than 0.05 considered statistically significant. Effect sizes for 2-group comparisons were estimated using Cohen’s *d*, with the following thresholds applied: a Cohen’s *d* of ≤0.2 indicated a small effect, 0.5 a medium effect, and ≥0.8 a large effect (97). For comparisons involving multiple groups, 1-way ANOVA was used, followed by Tukey’s honestly significant difference or Dunnett’s multiple-comparison post hoc test for comparisons with the control group. Effect sizes for multiple-group comparisons were estimated using η^2^, with thresholds defined as follows: η^2^ ≤ 0.01 indicated a small effect, η^2^ = 0.06 a medium effect, and η^2^ ≥ 0.14 a large effect (97). A total of 3 wells in the *AP4B1^KO^* plus *NLRP5* control group were excluded from analysis as a result of screening artifacts.

The morphological profile of the TGN was analyzed using TGN roughness (shape factor in the MetaXpress software) and the following calculated metrics:
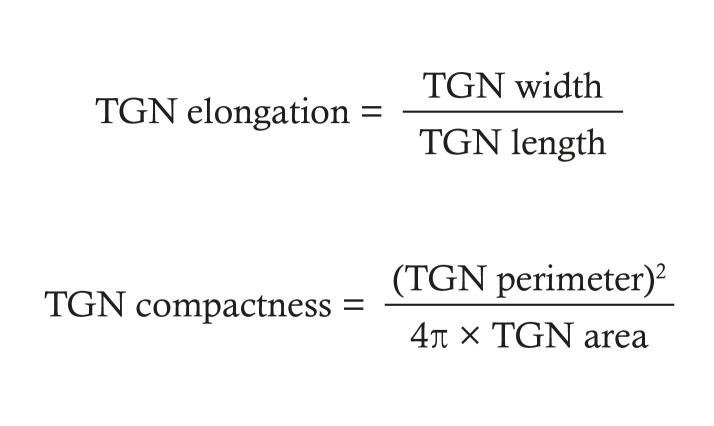


For pathway analysis, a list of GO terms associated with genes from the screen was derived from Ensembl (Supplemental File 3). GO terms with less than 3 or more than 1,000 genes in our screen were excluded. For each GO term, a pathway impact score was calculated (30):
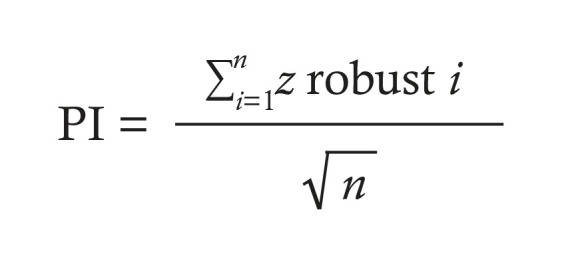
where PI is pathway impact score, *n* is number of genes associated with GO term, and *z* robust *i* is robust *z* score for each gene associated with GO term. The pathway impact (PI) score calculation for each GO term size, i.e., the number of genes comprised by each GO term, was performed 10,000 times using randomly selected values from the screen for the robust *z* score. From this, the normal distribution of PI scores for each GO term could be derived and the probability *P* of obtaining the actual PI score by chance was calculated. Similar-sized GO terms yielded a similar normal distribution. Therefore, *P* values were adjusted for multiple testing according to Bonferroni by multiplication by the number of occurrences of the respective GO term size. A pathway was considered significantly affected if adjusted *P* was less than 0.05.

To determine the overlap between 2 pathways, we calculated the overlap coefficient (30):
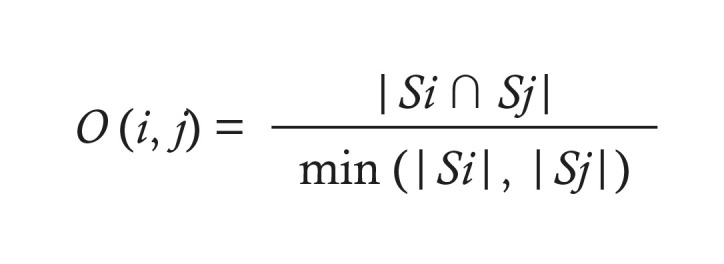
where |*Si* ∩ *Sj*| is the number of shared genes between the 2 gene sets *Si* and *Sj*, and min (|*Si*|, |*Sj*|) is the size of the smaller set.

A matrix of overlap coefficients was calculated for all pathways. From this, a distance matrix based on Euclidean distance was generated, followed by average-linkage clustering. The hierarchical clustering order was applied to reorder the overlap coefficient matrix, and a heatmap view was created for visualization. Clusters of highly overlapping pathways were identified using the Dynamic Tree Cut algorithm (83). Suitable umbrella terms were manually assigned to each cluster to provide descriptive labels. For the proteins associated with each cluster, family domain information was retrieved from the Universal Protein Resource (UniProt) (82).

Plotting and statistical analysis for Supplemental Figure 5 were performed using GraphPad Prism version 10 (GraphPad Software).

The Synthego sgRNA Human Druggable Genome library is available through the ICCB-Longwood Screening Facility at Harvard Medical School (https://iccb.med.harvard.edu/synthego-sgrna-human-druggable-genome). For the detailed nucleofection protocol for the Lonza 4D-Nucleofector, see https://bioscience.lonza.com/lonza_bs/DE/en/document/21324

### Study approval

No human or animal studies were conducted.

### Data availability

Screening data were deposited at Zenodo and are publicly available (doi:10.5281/zenodo.10574989). Relevant original code was deposited at Zenodo and is publicly available (doi:10.5281/zenodo.10574989). Any additional information required to reanalyze the data reported in this paper is available upon request. Source data for figures are available in the Supporting Data Values file. Requests for further information and for resources and reagents should be directed to and will be fulfilled by the corresponding author.

## Author contributions

MZ, CG, JEA, XX, AS, AKD, and DEF conceptualized and designed the experiments. MZ, CG, JEA, XX, HMK, and DEF performed experiments. MZ and DEF wrote the first draft of the manuscript. All authors contributed to the final draft of the manuscript. DEF wrote the grants that designed and supported this project. DEF and MS supervised the project. CG and JEA contributed equally to this work. CG is listed first in recognition of contributions made at later stages of the project.

## Conflict of interest

DEF serves on the scientific advisory board (unpaid) for the following foundations: CureAP4 Foundation, the Maddie Foundation, the SPG69/Warburg Micro Research Foundation, the Lilly & Blair Foundation, the Maurya Koduri Foundation, and Genetic Cures for Kids Inc. DEF received speaker honoraria from the International Parkinson and Movement Disorders Society and publishing royalties from Cambridge University Press. DEF and MS hold the following patent: PCT/US2024/029856.

## Funding support

This work is the result of NIH funding, in whole or in part, and is subject to the NIH Public Access Policy. Through acceptance of this federal funding, the NIH has been given a right to make the work publicly available in PubMed Central.

- CureAP4 Foundation (to DEF).
- Spastic Paraplegia Foundation (to DEF).
- Manton Center for Orphan Disease Research (to DEF).
- Boston Children’s Hospital (BCH) Office of Faculty Development (to DEF).
- BCH Translational Research Program (to DEF).
- National Institute of Neurological Disorders and Stroke, grants 1K08NS123552-01 and 1U54NS148312 (to DEF).
- German National Academic Foundation (to MZ, CG, and JEA).
- Bayer Foundation, Carl Duisberg Fellowship for Medical Sciences (to MZ).
- German National Exchange Service, Biomedical Education Program (to MZ, CG, and JEA).
- RWTH Aachen Research Ambassador Scholarship (to CG).
- German Research Foundation, grant 448402208 (to AS).
- European Union’s Horizon 2020 research and innovation program, Marie Skłodowska-Curie grant agreement 896725 (to AKD).
- Rosamund Stone Zander chair (to MS).
- National Institutes of Health, grant 1U54HD090255 (to the Intellectual and Developmental Disabilities Research Center at Boston Children’s Hospital).

## Supplementary Material

Supplemental data

Supplemental data set 1

Supplemental data set 2

Supplemental data set 3

Supplemental data set 4

Supplemental data set 5

Supplemental data set 6

Unedited blot and gel images

Supporting data values

## Figures and Tables

**Table 1:**
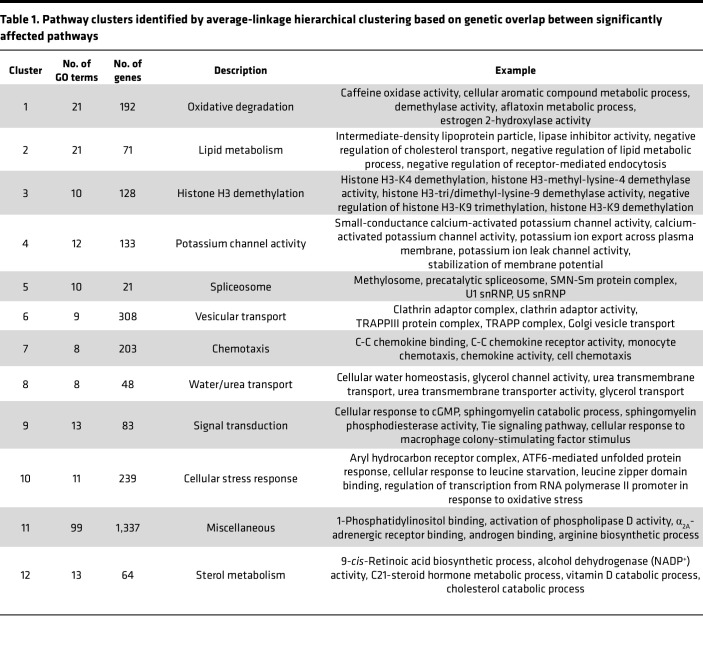
Pathway clusters identified by average-linkage hierarchical clustering based on genetic overlap between significantly affected pathways

## Version 1 05/19/2026

In-Press Preview

## Version 2 07/22/2026

Electronic publication
