# Supplementary material for: CRISPR/Cas9 loss-of-function screen in a neuronal model of AP-4 deficiency identifies ATG9A trafficking modulators
Source: JCI Insight. 2026 May 19;11(14):e202204. doi: 10.1172/jci.insight.202204 (PMC13461165; doi:10.1172/jci.insight.202204)
Supplement: Supplementary file 1 [file jciinsight-11-202204-s084.pdf]

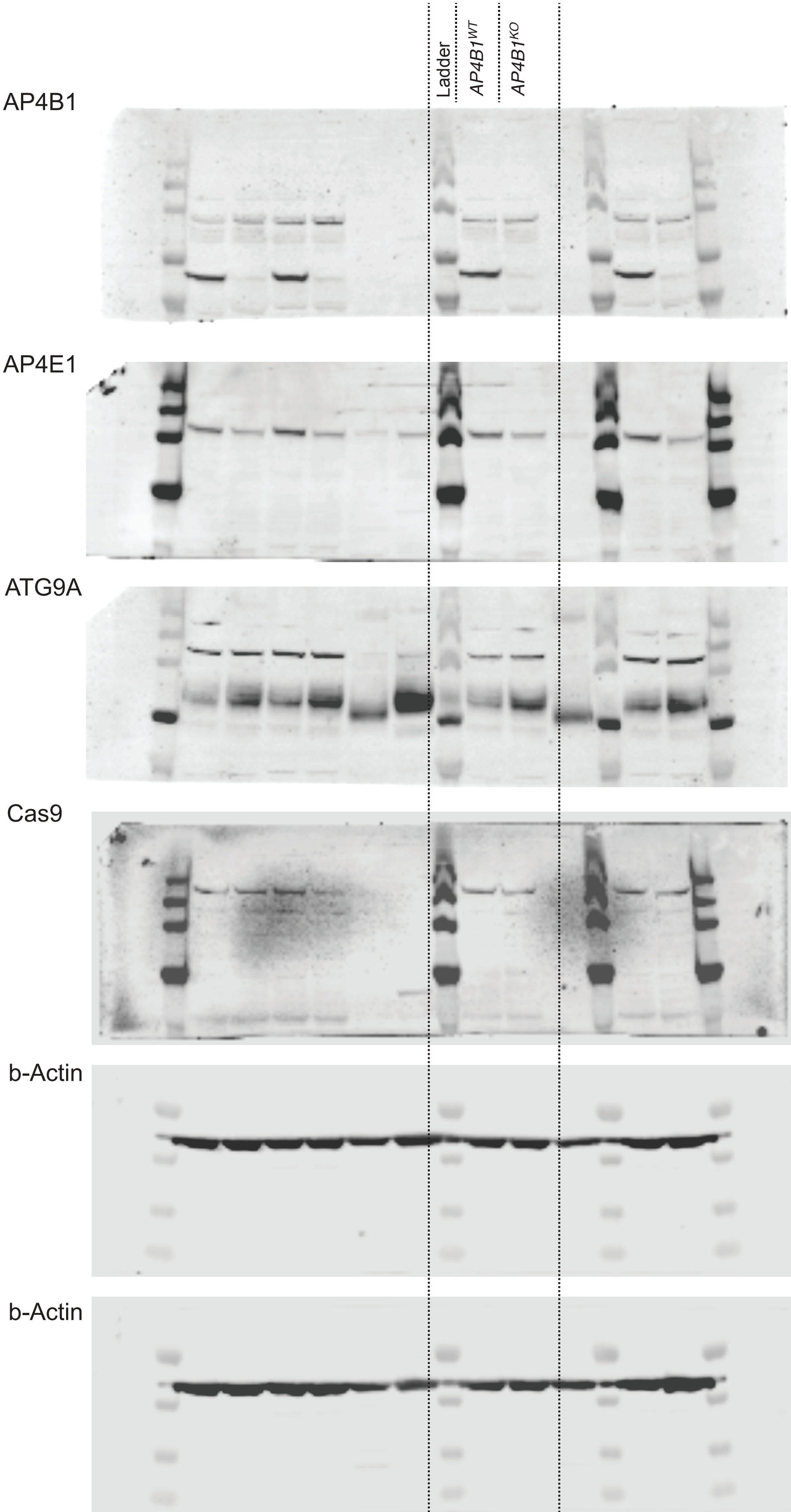

Ladder
AP4B1^WT
AP4B1^KO
AP4B1
AP4E1
ATG9A
Cas9
b-Actin
b-Actin

**Supplementary Figure 1: Uncut Western Blots (*Figure 1B*)**

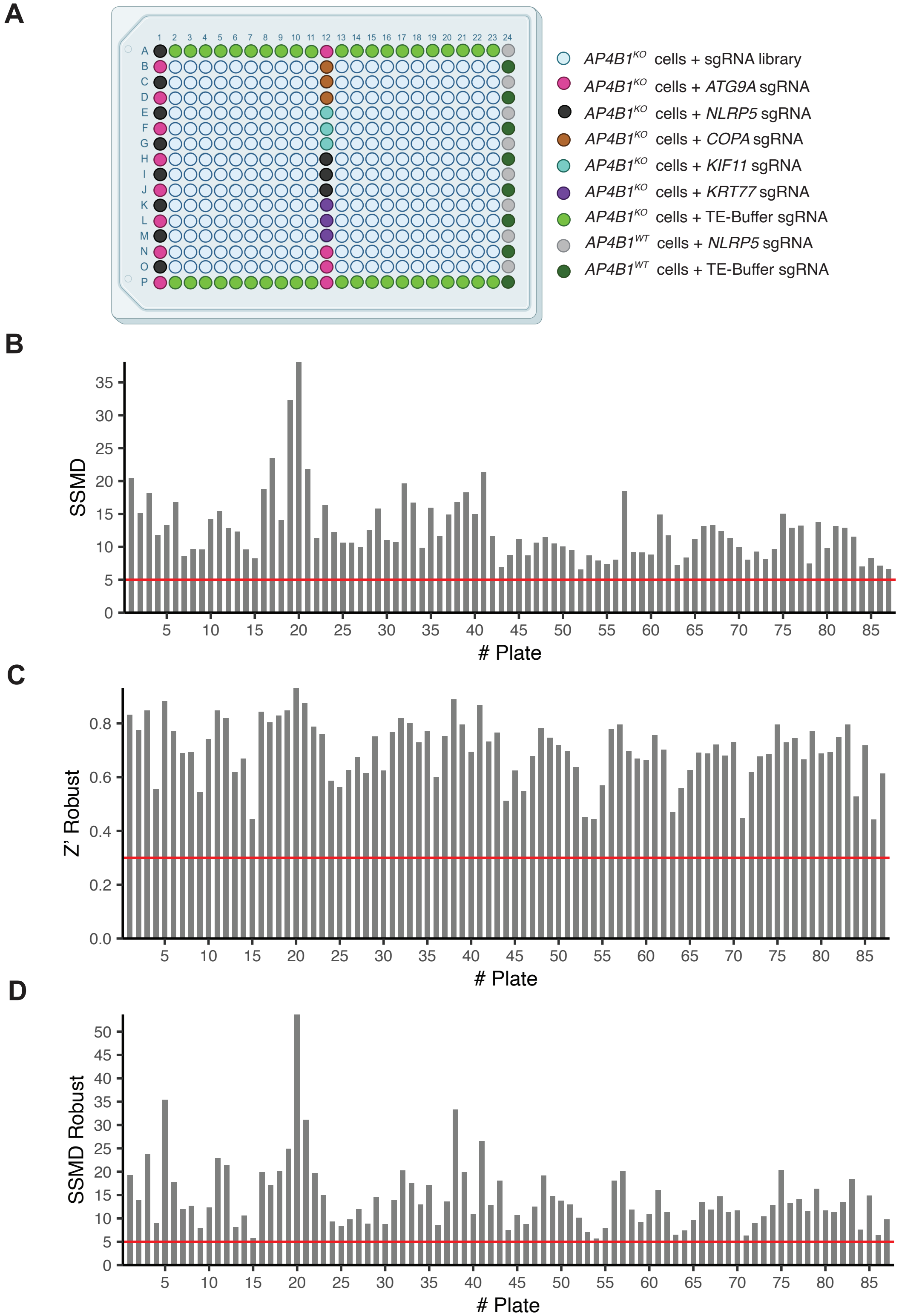
A
B
C
D
AP4B1^KO cells + sgRNA library
AP4B1^KO cells + ATG9A sgRNA
AP4B1^KO cells + NLRP5 sgRNA
AP4B1^KO cells + COPA sgRNA
AP4B1^KO cells + KIF11 sgRNA
AP4B1^KO cells + KRT77 sgRNA
AP4B1^KO cells + TE-Buffer sgRNA
AP4B1^WT cells + NLRP5 sgRNA
AP4B1^WT cells + TE-Buffer sgRNA
SSMD
Z' Robust
SSMD Robust
# Plate

**Supplementary Figure 2: Primary Screen Plate Design and Quality Control Metrics**

*A)* Plate layout for primary screen. Positive controls included *AP4B1*$^{KO}$ cells transfected with sgRNAs targeting *ATG9A*, and *AP4B1*$^{WT}$ cells treated with TE-buffer or transfected with sgRNAs targeting the non-essential gene *NLRP5*. Negative controls comprised *AP4B1*$^{KO}$ cells treated with TE-buffer or transfected with sgRNAs targeting non-essential genes *NLRP5* or *KRT77*. To assess transfection and knockout efficiency, cell death-inducing sgRNAs targeting essential genes *COPA* and *KIF11* were used. This figure was partially created in BioRender. Kim, H. (2026) https://BioRender.com/hiargyc.

*B)* Strictly standardized mean difference (SSMD) for all 87 screened plates demonstrated robust assay performance. Horizontal line indicates predefined quality control threshold (SSMD > 5).

*C)* Z' robust for all 87 screened plates. Horizontal line indicates predefined quality control threshold (Z' > 0.3).

*D)* SSMD robust for all 87 screened plates. Horizontal line represents predefined quality control threshold (SSMD robust > 5).

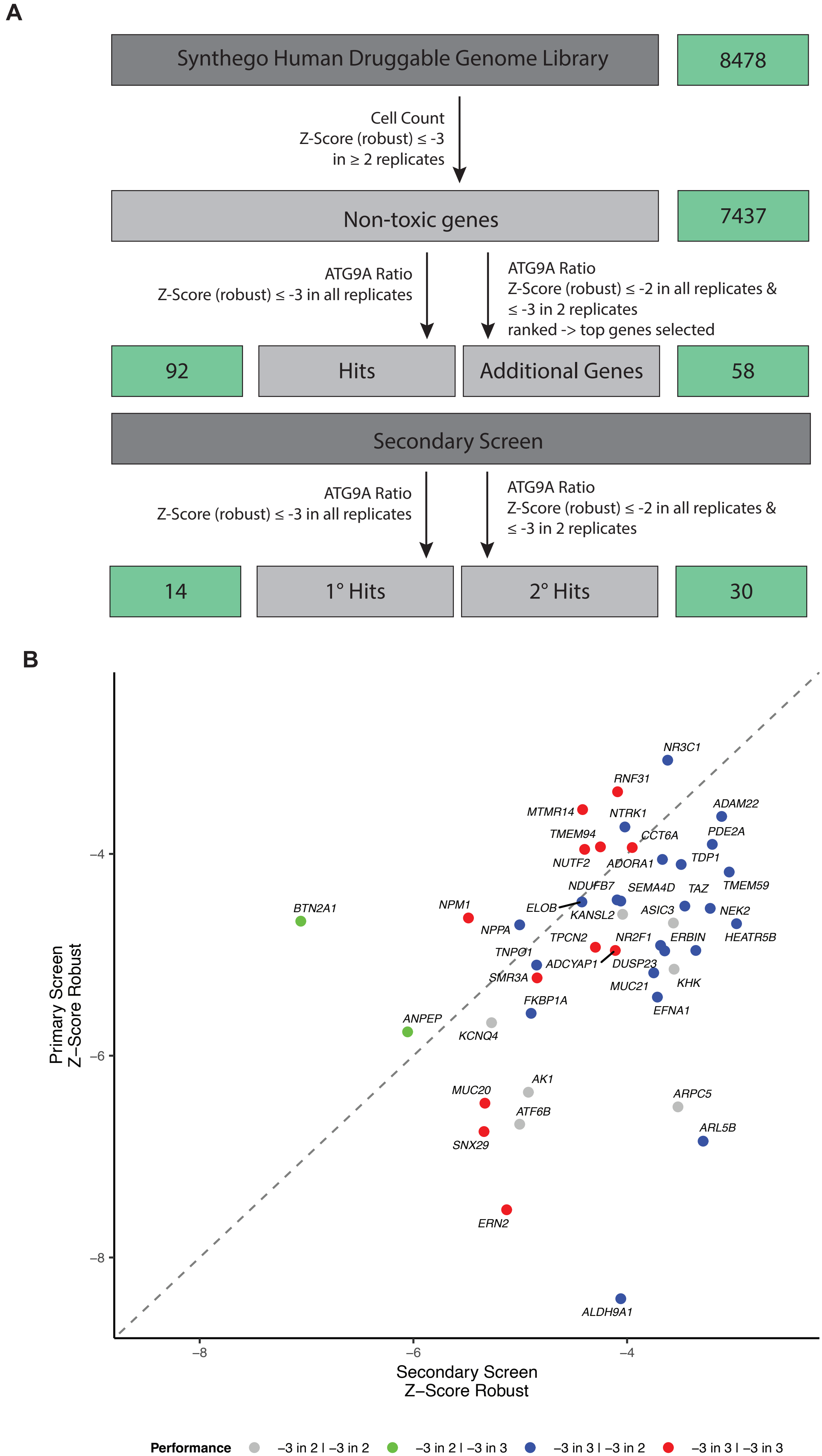
A
Synthego Human Druggable Genome Library
8478
Cell Count
Z-Score (robust) ≤ -3
in ≥ 2 replicates
Non-toxic genes
7437
ATG9A Ratio
Z-Score (robust) ≤ -3 in all replicates
ATG9A Ratio
Z-Score (robust) ≤ -2 in all replicates &
≤ -3 in 2 replicates
ranked -> top genes selected
92
Hits
Additional Genes
58
Secondary Screen
ATG9A Ratio
Z-Score (robust) ≤ -3 in all replicates
ATG9A Ratio
Z-Score (robust) ≤ -2 in all replicates &
≤ -3 in 2 replicates
14
1° Hits
2° Hits
30
B
Primary Screen
Z–Score Robust
Secondary Screen
Z–Score Robust
NR3C1
RNF31
MTMR14
NTRK1
ADAM22
TMEM94
CCT6A
PDE2A
NUTF2
ADORA1
TDP1
NDUFB7
SEMA4D
TAZ
TMEM59
BTN2A1
NPM1
ELOB
KANSL2
ASIC3
NEK2
NPPA
HEATR5B
TPCN2
NR2F1
ERBIN
TNPO1
ADCYAP1
DUSP23
SMR3A
MUC21
KHK
FKBP1A
EFNA1
ANPEP
KCNQ4
AK1
MUC20
ARPC5
ATF6B
ARL5B
SNX29
ERN2
ALDH9A1
Performance
–3 in 2 I –3 in 2
–3 in 2 I –3 in 3
–3 in 3 I –3 in 2
–3 in 3 I –3 in 3

**Supplementary Figure 3: Overview of Primary and Secondary Screens and Comparison of Hit Genes**

*A)* Schematic overview of hit identification strategy for the primary and secondary screen. The ‘Synthego Human Druggable Genome’ sgRNA library targeted 8,478 genes. Toxic sgRNAs were excluded based on reduction of cell count by at least 3 standard deviations in two or more replicates, resulting in 7,437 non-toxic genes. Non-toxic genes were ranked according to ATG9A ratio reduction. A total of 92 genes reduced ATG9A ratio with a z-score robust < -3 in all replicates and were classified as hits. For secondary screening, additional 58 genes were selected from the top of the list of genes with a z-score robust < -3 in two replicates and < -2 in the third replicate, ranked by their mean ATG9A ratio across all replicates.

*B)* Comparison of confirmed hits from the secondary screen with primary screen results. Data are presented as mean z-scores robust across all replicates in the respective screen. Colors indicate if z-score robust of < -3 was achieved in only two replicates or across all three replicates.

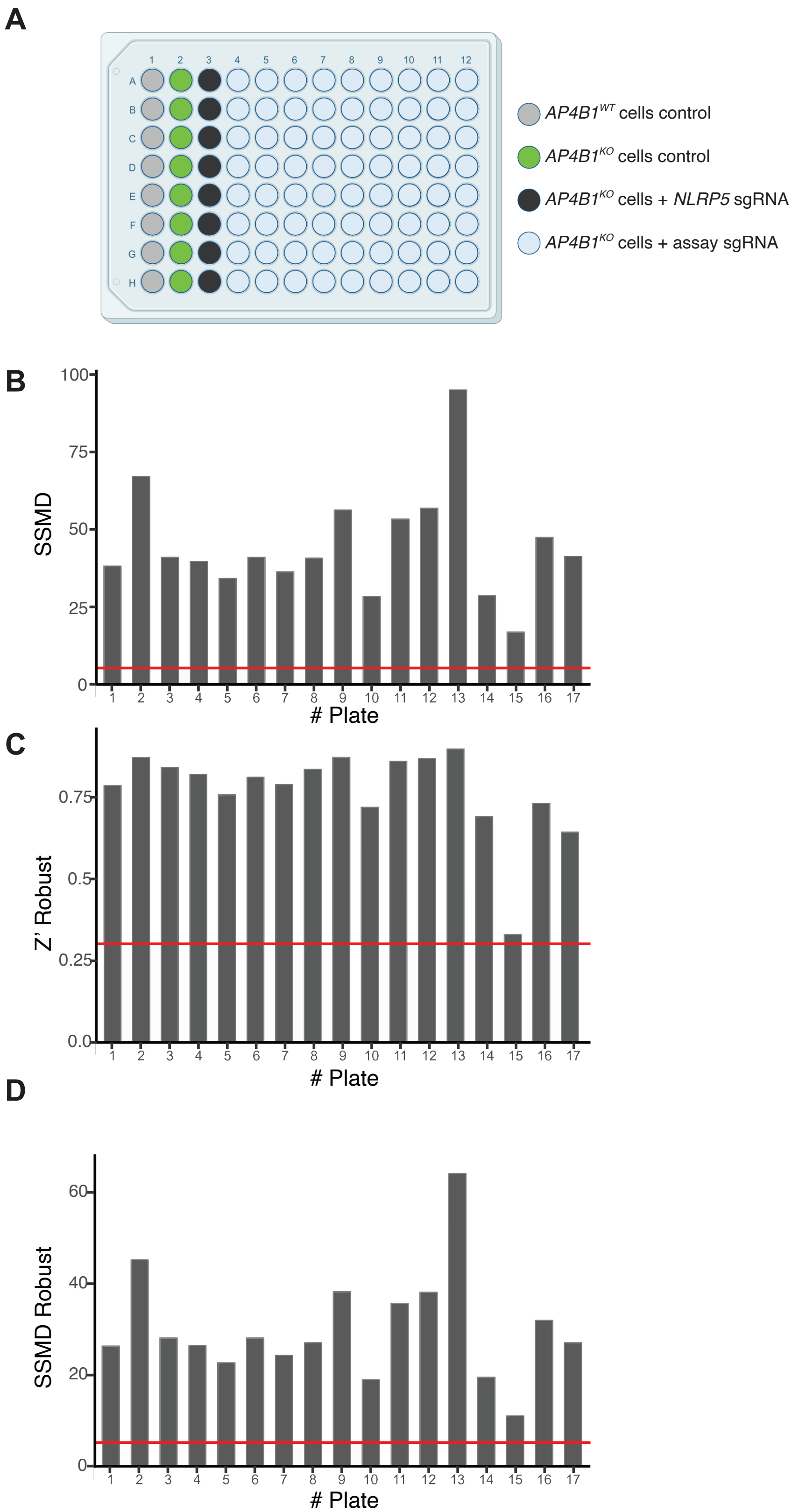

A
AP4B1^WT cells control
AP4B1^KO cells control
AP4B1^KO cells + NLRP5 sgRNA
AP4B1^KO cells + assay sgRNA
B
SSMD
# Plate
C
Z' Robust
# Plate
D
SSMD Robust
# Plate

**Supplementary Figure 4: Validation Experiments Plate Design and Quality Control Metrics**

A) Plate layout for validation experiments. Untransfected *AP4B1*$^{WT}$ cells served as positive control. Negative controls consisted of *AP4B1*$^{KO}$ cells untransfected or with transfection of sgRNAs targeting the non-essential gene *NLRP5*. This figure was partially created in BioRender. Kim, H. (2026) https://BioRender.com/7ig5l8j.

B) Strictly standardized mean difference (SSMD) for all 17 plates of the validation experiments. Horizontal line indicates the predefined quality control threshold (SSMD > 5).

C) Z'-factor robust for all 17 plates of the validation experiments. The horizontal line indicates the predefined quality control threshold (Z' > 0.3).

D) SSMD robust for all 17 plates of the validation experiments. Horizontal line represents predefined quality control threshold (SSMD robust > 5).

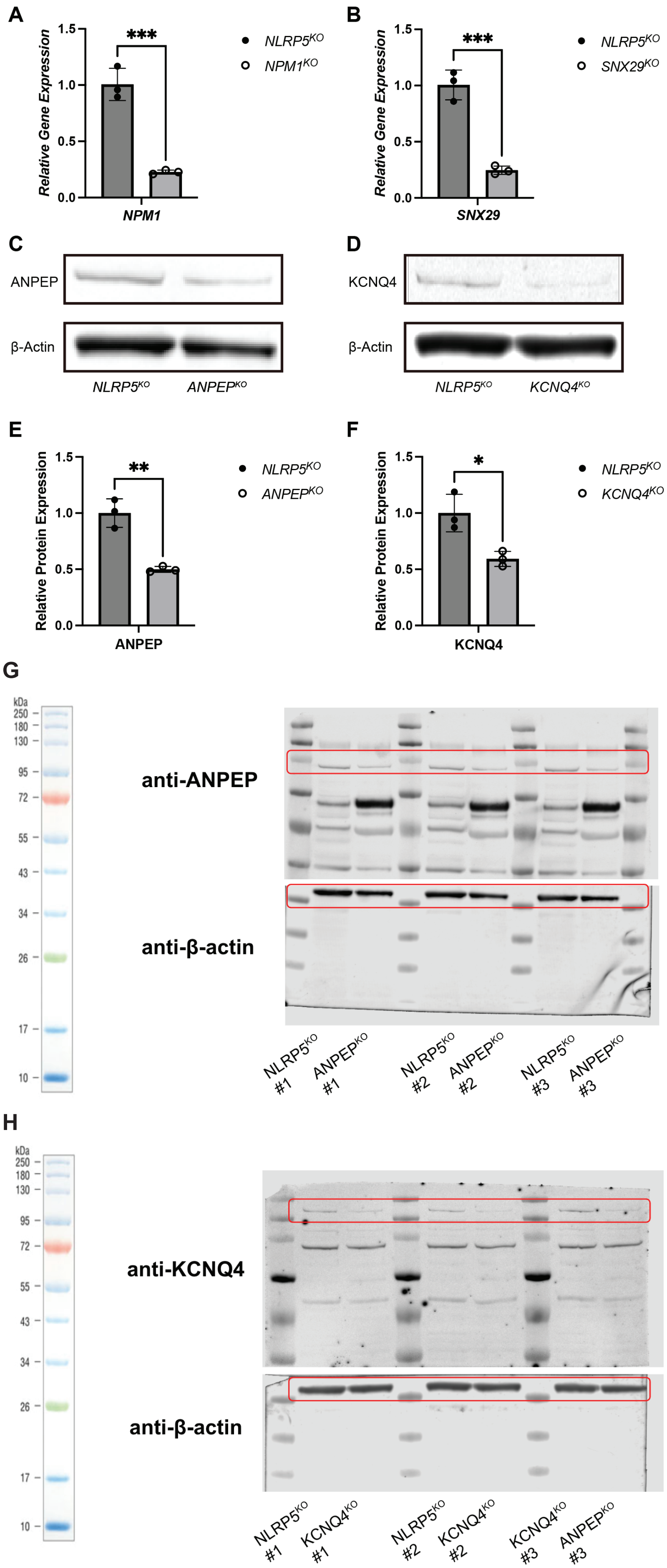
A
Relative Gene Expression
1.5
1.0
0.5
0.0
***
NPM1
NLRP5KO
NPM1KO
B
Relative Gene Expression
***
SNX29
NLRP5KO
SNX29KO
C
ANPEP
β-Actin
NLRP5KO
ANPEPKO
D
KCNQ4
β-Actin
NLRP5KO
KCNQ4KO
E
Relative Protein Expression
**
ANPEP
NLRP5KO
ANPEPKO
F
Relative Protein Expression
*
KCNQ4
NLRP5KO
KCNQ4KO
G
kDa
250
180
130
95
72
55
43
34
26
17
10
anti-ANPEP
anti-β-actin
NLRP5KO #1
ANPEPKO #1
NLRP5KO #2
ANPEPKO #2
NLRP5KO #3
ANPEPKO #3
H
kDa
250
180
130
95
72
55
43
34
26
17
10
anti-KCNQ4
anti-β-actin
NLRP5KO #1
KCNQ4KO #1
NLRP5KO #2
KCNQ4KO #2
KCNQ4KO #3
ANPEPKO #3

**Supplementary Figure 5: Validation of knockout efficiency for selected hit genes.**

(A–B) Quantitative RT–PCR analysis demonstrating efficient depletion of (A) NPM1 and (B) SNX29 transcripts following gene knockout. Expression levels were normalized to RPL32 and are shown relative to control (NLRP5 knockout).

(C–D) Representative Western blot analyses showing reduced protein levels of (C) ANPEP and (D) KCNQ4 in corresponding knockout conditions compared to NLRP5 control, with β-actin as loading control.

(E–F) Densitometric quantification of (E) ANPEP and (F) KCNQ4 protein levels from Western blots in (C–D), normalized to β-actin and expressed relative to control.

(G–H) Full-length Western blots for (G) ANPEP (G) and (H) KCNQ4, including replicate samples, confirming specificity and consistency of protein depletion.

All data represent three independent biological replicates. Statistical significance is indicated as $p < 0.05$ (), $p < 0.01$ (), and $p < 0.001$ ().

| REAGENT or RESOURCE | SOURCE | IDENTIFIER |
|---|---|---|
| **Antibodies** | | |
| Donkey anti-Sheep IgG (H+L) Cross-Adsorbed Secondary Antibody, Alexa Fluor™ 594 | Thermo Fisher Scientific | Cat# A-11016 |
| Goat anti-Mouse IgG (H+L) Cross-Adsorbed Secondary Antibody, Alexa Fluor™ 647 | Thermo Fisher Scientific | Cat# A-21235 |
| Goat anti-Rabbit IgG (H+L) Cross-Adsorbed Secondary Antibody, Alexa Fluor™ 488 | Thermo Fisher Scientific | Cat# A-11008 |
| IRDye® 680LT Donkey anti-Mouse IgG | LI-COR | Cat# 926-68022 |
| IRDye® 800CW Donkey anti-Rabbit IgG | LI-COR | Cat# 926-32213 |
| Mouse anti-AP4E1 | BD Biosciences | Cat# 612019 |
| Mouse anti-b-Actin | Sigma-Aldrich | Cat# A1978-100UL |
| Mouse Anti-CRISPR-Cas9 antibody | Abcam | Cat# ab191468 |
| Mouse Monoclonal Anti-β-Tubulin III | Sigma-Aldrich | Cat# T8660 |
| Rabbit anti-AP4B1 | Cell Signaling Technology | Cat# 26081 |
| Rabbit Anti-ATG9A antibody [EPR2450(2)] | Abcam | Cat# ab108338 |
| Rabbit anti-ANPEP | Abcam | Cat# ab108310 |
| Rabbit anti-KCNQ4 | A gift from Dr. Bechara Kachar, NIDCD, Bethesda, MD USA | |
| Sheep anti Human TGN46 | BioRad | Cat# AHP500G |
| **Chemicals, peptides, and recombinant proteins** | | |
| all-trans retinoic acid | MedChemExpress | Cat# HY-14649 |
| B-27™ Supplement (50X), serum free | Thermo Fisher Scientific | Cat# 17504044 |
| Bovine serum albumin | American Bioanalytical | Cat# AB01088-00100 |
| cOmplete™, EDTA-free Protease Inhibitor Cocktail | Roche | Cat# 04693132001 |
| DMEM-F12 | Thermo Fisher Scientific | Cat# 10565-018 |
| DPBS | Thermo Fisher Scientific | Cat# 14190144 |
| Fetal Bovine Serum | Thermo Fisher Scientific | Cat# 10082147 |
| High-Capacity cDNA Reverse Transcription Kit | Thermo Fisher Scientific | Cat# 4368813 |

| | | |
|---|---|---|
| Hoechst 33258, Pentahydrate (bis-Benzimide) | Thermo Fisher Scientific | Cat# H3569 |
| Intercept® (TBS) Blocking Buffer | LI-COR | Cat# 927-60001 |
| Invitrogen™ Lipofectamine™ RNAiMAX Transfection Reagent | Thermo Fisher Scientific | Cat# 13778150 |
| L-Glutamine | Thermo Fisher Scientific | Cat# 25030081 |
| Laminin | Thermo Fisher Scientific | Cat# 23017015 |
| Methanol | Thermo Scientific | Cat# 268280025 |
| Neurobasal™ Medium | Thermo Fisher Scientific | Cat# 21103049 |
| NuPAGE™ LDS Sample Buffer (4X) | Thermo Fisher Scientific | Cat# NP0007 |
| NuPAGE™ MOPS SDS Running Buffer (20X) | Thermo Fisher Scientific | Cat# NP0001 |
| NuPAGE™ Sample Reducing Agent (10X) | Thermo Fisher Scientific | Cat# NP0009 |
| NuPAGE™ Transfer Buffer (20X) | Thermo Fisher Scientific | Cat# NP00061 |
| Opti-MEM™ Reduced Serum Media | Thermo Fisher Scientific | Cat# 31985070 |
| Paraformaldehyde Aqueous Solution 8% | Electron Microscopy Sciences | Cat# 157-8-100 |
| Penicillin/Streptomycin | Thermo Fisher Scientific | Cat# 15140122 |
| PhosSTOP™ | Roche | Cat# 04906837001 |
| RIPA Lysis and Extraction Buffer | Thermo Fisher Scientific | Cat# 89900 |
| RNeasy Mini Kit | Qiagen | Cat# 74106 |
| RNaseZap™ RNase Decontamination Solution | Thermo Fisher Scientific | Cat# AM9780 |
| Saponin | Sigma-Aldrich | Cat# 47036-250G-F |
| Sodium Dodecylsulfate Solution 20% | Thermo Fisher Scientific | Cat# J75832.AP |
| SYBR™ Green Universal Master Mix | Thermo Fisher Scientific | Cat# 4334973 |
| Trypsin-EDTA 0.25% | Thermo Fisher Scientific | Cat# 25200056 |
| TWEEN® 20 | Sigma-Aldrich | Cat# P9416 |
| **Critical commercial assays** | | |
| Pierce™ BCA Protein Assay Kit | Thermo Fisher Scientific | Cat# 23225 |
| **Deposited data** | | |
| Raw and analyzed data | This paper | *DOI:* 10.5281/zenodo.10574989 |
| R script | This paper | *DOI:* 10.5281/zenodo.10574989 |

| | | |
|---|---|---|
| **Experimental models: Cell lines** | | |
| SH-SY5Y | | Described in Davies et al.[10] |
| **Oligonucleotides** | | |
| *ANPEP* multiguide knockout kits (v2) sgRNA | Synthego | UUGGUGGUGGCUGAGGCGGA, ACUGUCAGUGGUGUACUCCC, GAGGAUCCCCAGGAUGCCCA |
| *ATG9A* multiguide knockout kits (v2) sgRNA | Synthego | UGAGCAGGCAAAAAGGCGUC, ACUGUGGUUCACCAUCUUGU, UAGUGAAGGCAACCACAAAG |
| *KCNQ4* multiguide knockout kits (v2) sgRNA | Synthego | ACAGGAAUUCGUGAUGAUCG, GUAGCGGCAGCAGCAUCCGG, CUCAUUACCGAUGACACAGA |
| *NLRP5* multiguide knockout kits (v2) sgRNA | Synthego | UGAAAUCGAGAAUGCCAACG, UUAUGGAGCAUCGCUGGCCU, GUGUCUCUAUGAGCUAGACA |
| *NPM1* multiguide knockout kits (v2) sgRNA | Synthego | AUUUACAGGUUGUGAACUAA, GUACCGUUCUUAAAGAUAAC, CCUUUUAGUUUCUAUUCAUG |
| *SNX29* multiguide knockout kits (v2) sgRNA | Synthego | UUCACCUUAGGCCACUGGAA, UCAUUGUGCAGCACCUUCAG, GCUCCUACAGCCCAGCAGGU |
| *ANPEP* Primer | IDT | *FWD:* CCTCCCCTTTGGGGATATAAGCC<br>*REV:* GGTGCTGGAGCCCTTAAAAACG |
| *ATG9A* Primer | IDT | *FWD:* CCTTCCAGTTCCCAGTCTTGTTC<br>*REV:* CAGAGACGCTGCTATCCTGAAG |
| *KCNQ4* Primer | IDT | *FWD:* CTCCGGAATCGTCAAGTCCAGG<br>*REV:* CAGAGTCGGGATTGGGAGTAAAG |
| *NLRP5* Primer | IDT | *FWD:* CTTGAGAATTTGCTGCAAGATCCT<br>*REV:* CGATTCTTCCCTGTTCCCATGAG |

| | | |
|---|---|---|
| *NPM1* Primer | IDT | *FWD:* GGTGTCTGTAGAGGTTTCTGTTC<br>*REV:* CCAAATGAGTGGTATAGATGCC |
| *SNX29* Primer | IDT | *FWD:* GCAAGAGGTCTGGATCTGGACTC<br>*REV:* CCTCTCTCACTTCTGCATCTCAG |
| *RPL32* Primer (for qPCR) | | *FWD:* CATCTCCTTCTCGGCATCA<br>*REV:* CTGGGTTTCCGCCAGTTAC |
| *NLRP5* Primer (for qPCR) | | *FWD:* CTGGACGCCTTCCACTGTCTTT<br>*REV:* CGCAAATACGGACAGTGCTGGA |
| *NPM1* Primer (for qPCR) | | *FWD:* GGCCGACAAAGATTATCACT<br>*REV:* CTGCCTTCGTAATTCATTGCC |
| *SNX29* Primer (for qPCR) | | *FWD:* AGGAGATTGCCTCGGATTCCGA<br>*REV:* AGTGCCAATCCTCGACTCCTCT |
| **Software and algorithms** | | |
| BioRender | https://www.biorender.com | |
| Synthego Inference of CRISPR Edits (ICE) Tool | https://ice.editco.bio/#/ | |
| Image Studio | LI-COR | 5.2.5 |
| IUPHAR/BPS Guide to PHARMACOLOGY | https://www.guidetopharmacology.org<br>(accessed: 02/18/2025) | |
| MetaXpress | Molecular Devices | 6.7.1.157 |
| PANTHER (Protein ANalysis THrough Evolutionary Relationships) Classification System | https://www.pantherdb.org<br>(accessed: 09/19/2022) | |
| R | Open Source | 4.2.0 |
| RStudio | RStudio | 2022.12.0+353 |
| **Other** | | |
| AbsorbMax™ Sealing Films | Excel Scientific | Cat# BK-50 |
| Cell Culture Microplate, 384 Well, PS, F-Bottom | Greiner | Cat# 781090 |
| Cell Lifter | BIOLOGIX | Cat# 70-2180 |
| Countess™ Cell Counting Chamber Slides | Thermo Fisher Scientific | Cat# C10228 |
| Countess™ II Automated Cell Counter | Thermo Fisher Scientific | Cat# AMQAX1000 |

| | | |
|---|---|---|
| Eppendorf twin.tec® PCR Plate 384, skirted, 45 µL, PCR clean, green | Eppendorf | Cat# 951020729 |
| Greiner Bio-One 384w Cellstar Plate, Ps, Tc Treated, Sterile | Greiner Bio-One | Cat# 781090 |
| Greiner Bio-One 96w Sensoplate microplate, Sterile | Greiner Bio-One | Cat# 655892 |
| Immobilon®-FL PVDF Membrane | Millipore | Cat# IPFL00010 |
| Multidrop™ Combi 836 Reagent Dispenser | Thermo Fisher Scientific | Cat# 5840300 |
| NuPAGE™ 12%, Bis-Tris, 1.0 mm, Mini Protein Gels | Thermo Fisher Scientific | Cat# NP0342BOX |
| NuPAGE™ 4 to 12%, Bis-Tris, 1.0–1.5 mm, Mini Protein Gels | Thermo Fisher Scientific | Cat# NP0321BOX |
| Steriflip® Sterile Centrifuge Tube Top Filter Unit | Sigma-Aldrich | Cat# SCGP00525 |
| Tissue Culture Plate 6 Well, Sterile | Celltreat | Cat# 229106 |

Supplementary Table 1. Key Resources
