# Supplementary material for: CRISPR/Cas9 loss-of-function screen in a neuronal model of AP-4 deficiency identifies ATG9A trafficking modulators
Source: JCI Insight. 2026 May 19;11(14):e202204. doi: 10.1172/jci.insight.202204 (PMC13461165; doi:10.1172/jci.insight.202204)
Supplement: Supplementary file 2 [file jciinsight-11-202204-s091.pdf]

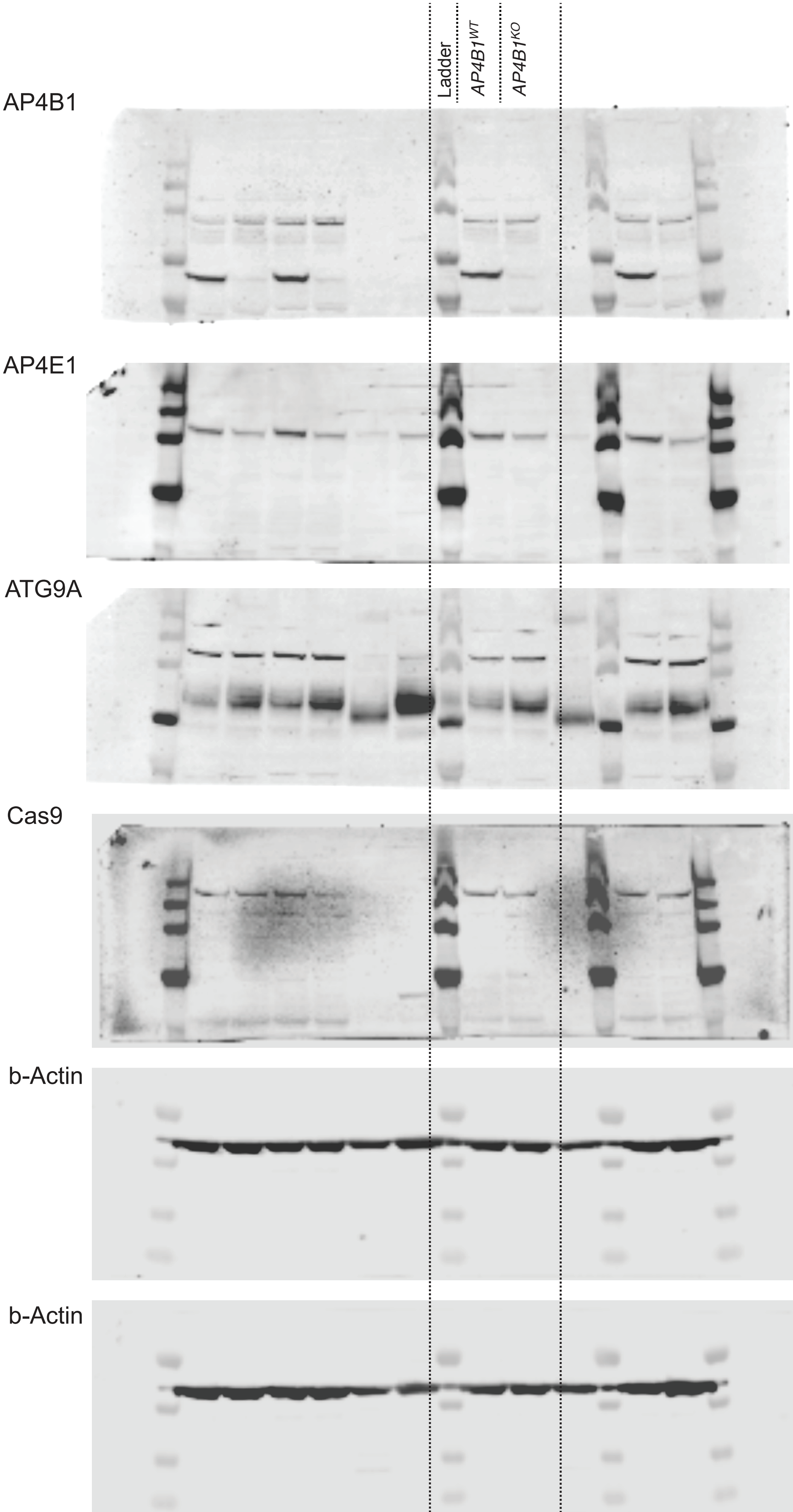
Ladder
AP4B1WT
AP4B1KO
AP4B1
AP4E1
ATG9A
Cas9
b-Actin
b-Actin

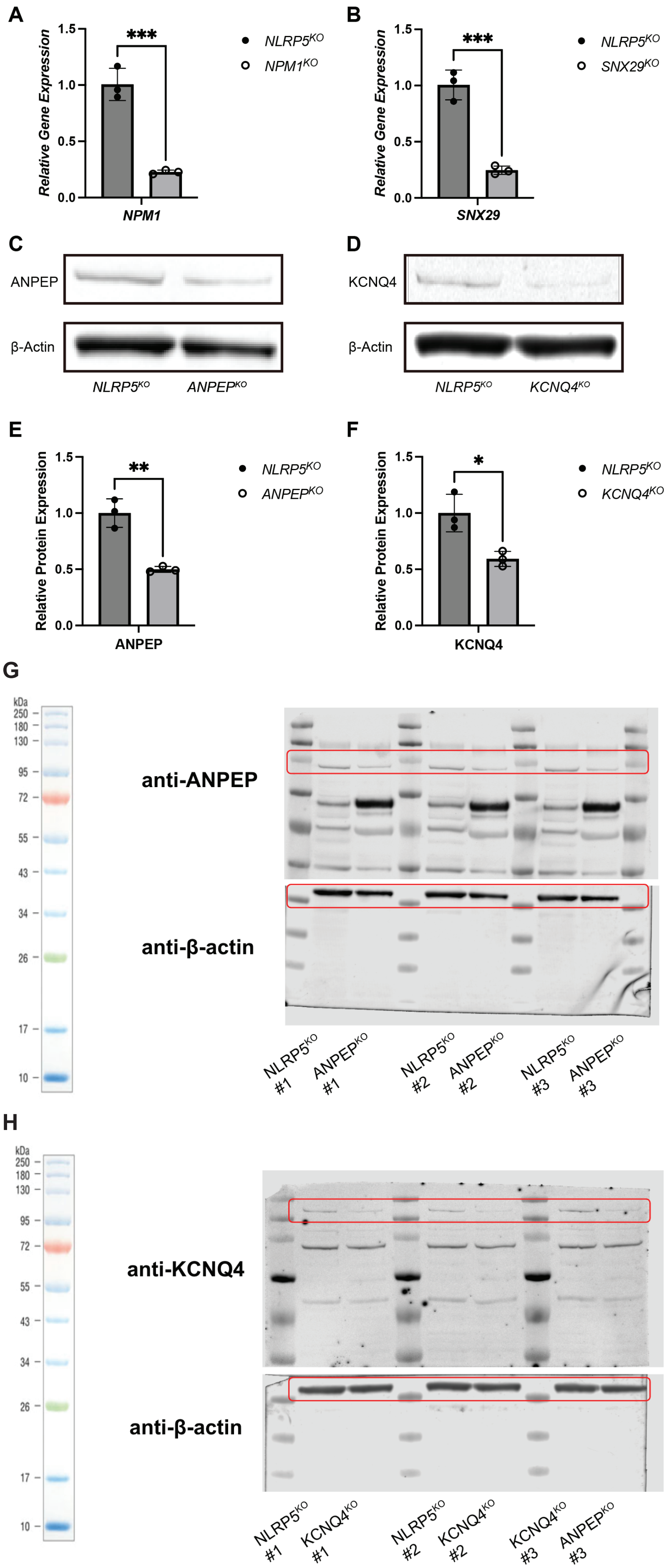

A
Relative Gene Expression
NPM1
NLRP5KO
NPM1KO
***
B
Relative Gene Expression
SNX29
NLRP5KO
SNX29KO
***
C
ANPEP
β-Actin
NLRP5KO
ANPEPKO
D
KCNQ4
β-Actin
NLRP5KO
KCNQ4KO
E
Relative Protein Expression
ANPEP
NLRP5KO
ANPEPKO
**
F
Relative Protein Expression
KCNQ4
NLRP5KO
KCNQ4KO
*
G
kDa
250
180
130
95
72
55
43
34
26
17
10
anti-ANPEP
anti-β-actin
NLRP5KO #1
ANPEPKO #1
NLRP5KO #2
ANPEPKO #2
NLRP5KO #3
ANPEPKO #3
H
kDa
250
180
130
95
72
55
43
34
26
17
10
anti-KCNQ4
anti-β-actin
NLRP5KO #1
KCNQ4KO #1
NLRP5KO #2
KCNQ4KO #2
KCNQ4KO #3
ANPEPKO #3
