# Supplementary material for: CRISPR/Cas9 loss-of-function screen in a neuronal model of AP-4 deficiency identifies ATG9A trafficking modulators
Source: JCI Insight. 2026 May 19;11(14):e202204. doi: 10.1172/jci.insight.202204 (PMC13461165; doi:10.1172/jci.insight.202204)
Supplement: Supplementary file 3 [file jciinsight-11-202204-s089.docx]

| **Cluster** | **Gene Ontology ID** | **Gene Ontology Name** |
| --- | --- | --- |
| 2 | GO:0070328 | triglyceride homeostasis |
| 3 | GO:0006325 | chromatin organization |
| 3 | GO:0006338 | chromatin remodeling |
| 4 | GO:0006813 | potassium ion transport |
| 4 | GO:0071805 | potassium ion transmembrane transport |
| 4 | GO:0008076 | voltage-gated potassium channel complex |
| 4 | GO:0005249 | voltage-gated potassium channel activity |
| 4 | GO:0005267 | potassium channel activity |
| 4 | GO:0005251 | delayed rectifier potassium channel activity |
| 6 | GO:0006886 | intracellular protein transport |
| 6 | GO:0016192 | vesicle-mediated transport |
| 6 | GO:0015031 | protein transport |
| 8 | GO:0051289 | protein homotetramerization |
| 9 | GO:0019933 | cAMP-mediated signaling |
| 9 | GO:0036006 | cellular response to macrophage colony-stimulating factor stimulus |
| 9 | GO:0043116 | negative regulation of vascular permeability |
| 9 | GO:0043951 | negative regulation of cAMP-mediated signaling |
| 9 | GO:0071321 | cellular response to cGMP |
| 9 | GO:0097011 | cellular response to granulocyte macrophage colony-stimulating factor stimulus |
| 9 | GO:0008081 | phosphoric diester hydrolase activity |
| 9 | GO:0004114 | 3',5'-cyclic-nucleotide phosphodiesterase activity |
| 9 | GO:0004115 | 3',5'-cyclic-AMP phosphodiesterase activity |
| 9 | GO:0047555 | 3',5'-cyclic-GMP phosphodiesterase activity |
| 10 | GO:0036500 | ATF6-mediated unfolded protein response |
| 10 | GO:0090575 | RNA polymerase II transcription regulator complex |
| 10 | GO:0046982 | protein heterodimerization activity |
| 10 | GO:0035497 | cAMP response element binding |
| 12 | GO:0006081 | aldehyde metabolic process |
| 12 | GO:0016620 | oxidoreductase activity, acting on the aldehyde or oxo group of donors, NAD or NADP as acceptor |
| 12 | GO:0004029 | aldehyde dehydrogenase (NAD+) activity |
| 12 | GO:0043878 | glyceraldehyde-3-phosphate dehydrogenase (NAD+) (non-phosphorylating) activity |

**Supplementary Table 1:**

Full Gene Ontology Terms for Gene Ontology IDs displayed in *Figure 4A*.
